# Analysis of the expression patterns, subcellular localisations and interaction partners of *Drosophila* proteins using a *pigP* protein trap library

**DOI:** 10.1242/dev.111054

**Published:** 2014-10

**Authors:** Nick Lowe, Johanna S. Rees, John Roote, Ed Ryder, Irina M. Armean, Glynnis Johnson, Emma Drummond, Helen Spriggs, Jenny Drummond, Jose P. Magbanua, Huw Naylor, Bénédicte Sanson, Rebecca Bastock, Sven Huelsmann, Vitor Trovisco, Matthias Landgraf, Seymour Knowles-Barley, J. Douglas Armstrong, Helen White-Cooper, Celia Hansen, Roger G. Phillips, The UK Drosophila Protein Trap Screening Consortium, Kathryn S. Lilley, Steven Russell, Daniel St Johnston

**Affiliations:** 1 The Gurdon Institute, University of Cambridge, Tennis Court Road, Cambridge CB2 1QN, UK; 2 The Department of Biochemistry, University of Cambridge, Tennis Court Road, Cambridge CB2 1QR, UK; 3 The Department of Genetics, University of Cambridge, Downing Street, Cambridge CB2 3EH, UK; 4 The Department of Physiology, Development and Neuroscience, University of Cambridge, Downing Street, Cambridge CB2 3EH, UK; 5 The Department of Zoology, University of Cambridge, Downing Street, Cambridge CB2 3EJ, UK; 6 Institute for Adaptive and Neural Computation, University of Edinburgh, 10 Crichton Street, Edinburgh EH8 9AB, UK; 7 Cardiff School of Biosciences, The Sir Martin Evans Building, Museum Avenue, Cardiff CF10 3AX, UK; 8 Department of Genetics, University of Leicester, Adrian Building, University Road, Leicester LE1 7RH, UK; 9 Centre for Advanced Microscopy, University of Sussex, School of Life Sciences, John Maynard Smith Building, Falmer, Brighton and Hove BN1 9QG, UK

**Keywords:** Affinity purification, Cytoophidia, Live imaging, piggyBac, Protein trap

## Abstract

Although we now have a wealth of information on the transcription patterns of all the genes in the *Drosophila* genome, much less is known about the properties of the encoded proteins. To provide information on the expression patterns and subcellular localisations of many proteins in parallel, we have performed a large-scale protein trap screen using a hybrid *piggyBac* vector carrying an artificial exon encoding yellow fluorescent protein (YFP) and protein affinity tags. From screening 41 million embryos, we recovered 616 verified independent YFP-positive lines representing protein traps in 374 genes, two-thirds of which had not been tagged in previous *P* element protein trap screens. Over 20 different research groups then characterized the expression patterns of the tagged proteins in a variety of tissues and at several developmental stages. In parallel, we purified many of the tagged proteins from embryos using the affinity tags and identified co-purifying proteins by mass spectrometry. The fly stocks are publicly available through the Kyoto *Drosophila* Genetics Resource Center. All our data are available via an open access database (Flannotator), which provides comprehensive information on the expression patterns, subcellular localisations and *in vivo* interaction partners of the trapped proteins. Our resource substantially increases the number of available protein traps in *Drosophila* and identifies new markers for cellular organelles and structures.

## INTRODUCTION

Since the sequencing of the *Drosophila melanogaster* genome over a decade ago, considerable effort has gone into identifying the full complement of protein-coding genes encoded in the genome and characterizing their expression profiles in different tissues and at different developmental stages (Adams et al., 2000; Arbeitman et al., 2002; Celniker et al., 2002; Misra et al., 2002; Graveley et al., 2011). This rich annotation has been enormously enhanced by phenotypic analyses that take advantage of the large number of gene knockouts generated by the *Drosophila* Gene Disruption Project and the creation of genome-wide RNA interference libraries (Spradling et al., 1999; Bellen et al., 2004, 2011; Dietzl et al., 2007; Ni et al., 2009, 2011). In addition, large-scale *in situ* hybridization screens have revealed where and when genes are expressed during embryogenesis (Lécuyer et al., 2007). Together, these approaches provide a wealth of data on the structural organization and expression patterns of many *Drosophila* genes, but information on their protein products is more limited. This is largely because it is much more laborious to perform genome-wide studies on proteins, because analyses require a specific antibody or tagged transgenic line for each protein.

The identification of interaction partners can often provide information on the subcellular localisation and function of a protein, as most cellular processes are performed by networks or complexes of interacting proteins. Most *Drosophila* protein-protein interaction data has come from yeast two-hybrid screens, which can identify candidate protein-protein interactions on a genome-wide scale (Giot et al., 2003; Stanyon et al., 2004; Formstecher et al., 2005). These screens have the advantage that they are high throughput and can detect interactions between proteins that are of low abundance *in vivo*. Their drawback is that they also detect interactions that are unlikely to occur *in vivo* because the two proteins are not expressed in the same cells or they localise to distinct subcellular compartments. They also miss interactions that depend on specific protein modifications that do not occur in the context of the yeast cell.

An alternative approach uses affinity tags to purify proteins from *in vivo* samples followed by the identification of co-purifying proteins by mass spectrometry (Gavin et al., 2002; Ho et al., 2002; Aebersold and Mann, 2003). This technique can identify native protein complexes in their normal physiological environment and can therefore detect indirect interactions and interactions that depend on modifications; however, it also suffers from false positives due to proteins that bind non-specifically to the affinity purification matrices or affinity tags. Higher confidence data can be obtained by integrating results from both approaches, as protein interactions that are detected in both two-hybrid screens and affinity purifications are estimated to be five to ten times more likely to occur *in vivo* than those detected using only one method (von Mering et al., 2002). Affinity purifications have only been performed on a genome-wide scale in *S. cerevisiae*, where the affinity tags can easily be introduced into open reading frames by homologous recombination (Gavin et al., 2002; Ho et al., 2002; Krogan et al., 2006). Although this approach has proved to be too laborious to apply on a large scale in higher metazoans, tagged expression constructs have recently been used to identify the interaction partners of several thousand *Drosophila* proteins in a tissue-culture cell system (Guruharsha et al., 2011).

**Fig. 1.**
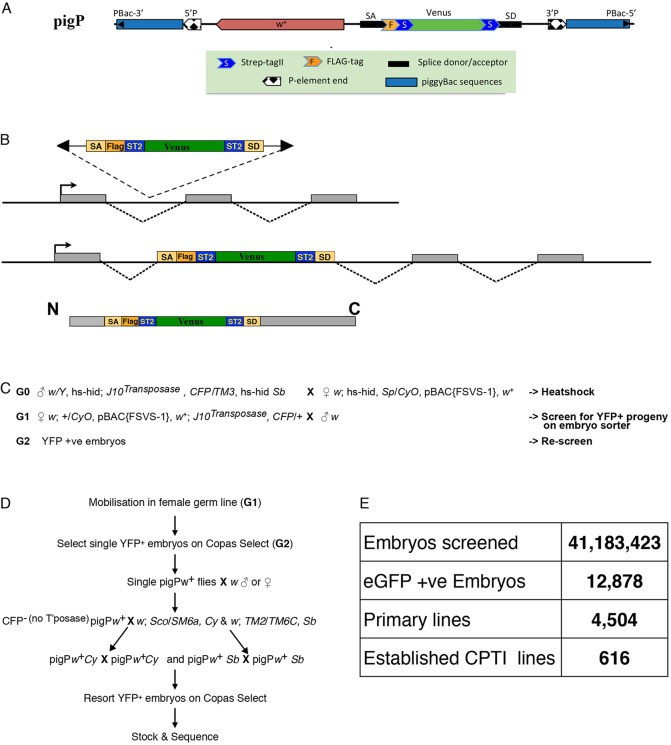
**Design of the pigP protein trap screen.** (A) Schematic of the *pigP* vector. (B) Schematic showing the inclusion of the affinity tags and Venus-YFP into the middle of a trapped protein after insertion of the *pigP* vector in the correct reading frame into an intron between protein-coding exons. (C) The crossing scheme used to generate *pigP* protein trap insertions. (D) Scheme for the recovery of YFP-positive *pigP* insertions. (E) Summary of the results of all *pigP* protein trap screens.

Here, we set out to introduce affinity tags into many different *Drosophila* proteins using the approach of protein trapping. This technique involves mobilizing a transposable element containing an artificial exon encoding a fluorescent marker, such as YFP, flanked by strong splice acceptor and donor sequences (Morin et al., 2001; Clyne et al., 2003). If the element inserts in the correct orientation into an intron between protein-coding exons in the appropriate reading frame, the YFP exon is spliced into the mature mRNA to produce a chimeric protein containing an internal YFP domain (Fig. 1A). YFP fluorescence can therefore be used to follow the expression and subcellular localisation of the protein in the living organism under its endogenous transcriptional and translational control. The two large-scale *Drosophila* protein trap screens that have been performed to date have generated 271 protein trap lines that have proved valuable tools for investigating protein localisation and function (Buszczak et al., 2007; Quinones-Coello et al., 2007). Based on the this success, we modified the protein trap strategy to introduce affinity tags into endogenous proteins along with the fluorescent reporter, so that we could perform pull-downs and mass spectrometry to identify their *in vivo* protein-interaction partners. Here, we report the results of this screen and the subsequent characterization of the expression patterns, subcellular localisations and interaction partners of the protein trap lines we identified.

## RESULTS

### Design of the screen

Previous protein trap screens have mainly used *P* element vectors, which have a strong bias towards inserting near the 5′ ends of genes and show many insertional hotspots (Bellen et al., 2004, 2011). We therefore chose to use a *piggyBac* vector, as this transposon has been reported to insert more randomly into the genome, at a consensus TTAA target site. Furthermore, screens indicate that ∼18% of *piggyBac* insertions map to introns within protein-coding regions (Hacker et al., 2003; Thibault et al., 2004). We originally generated constructs using the minimal *piggyBac* vector pXL-BacII to keep the size of the element as small as possible (Li et al., 2001). Although these constructs could be efficiently introduced into the *Drosophila* genome by germline transformation, we observed no transpositions or excisions from 4200 progeny when attempting construct mobilisations with ten independent starting lines. This suggests that internal *piggyBac* sequences missing from the minimal vectors are essential for efficient re-mobilisation of genomic *piggyBac* insertions, and we therefore used a full-length *piggyBac* vector (p3E1.2) for our subsequent constructs (Fraser et al., 1995). One difference between *piggyBac* and *P* element transposons is that the former almost invariably excise precisely, whereas *P* elements often undergo imprecise excisions that delete flanking sequences – a property that has proved extremely useful for generating loss of function alleles (Voelker et al., 1984; Daniels et al., 1985). We therefore included *P* element ends within our *piggyBac* vectors so that mutations in tagged genes can be subsequently generated by imprecise excision (Venken and Bellen, 2005). Within this hybrid *pigP* element, we introduced an artificial exon based on the construct designed by Morin et al. (2001), consisting of strong splice acceptor and donor sequences from the *Myosin heavy chain* (*Mhc*) locus flanking a YFP-Venus open reading frame fused to one or more protein affinity tags (Fig. 1A,B). We generated a set of 11 *pigP* vectors (supplementary Materials and Methods and Fig. S1A) that contained either two copies of StrepTagII and a 3×FLAG epitope fused in frame to the Venus-YFP-coding region in all three reading frames (FSVS vectors), or identical constructs with only two copies of StrepTagII (SVS vectors; supplementary material Fig. S1B). StrepTagII and FLAG tags have been reported to give lower background in pull-downs than other commonly used affinity tags and do not appear to affect the sub-cellular localisation of the proteins to which they are attached (supplementary Materials and Methods and http://www.flyprot.org/construct_notes.php) (Lichty et al., 2005). In addition to the affinity tags, YFP can also be efficiently pulled down from extracts using a GFP nanobody, and most trapped proteins therefore contain three different tags that can be used for affinity purifications (Rothbauer et al., 2008; Rees et al., 2011; Neumüller et al., 2012).

### Isolation of new protein trap lines

After a small pilot screen in which YFP-positive larvae were selected manually, we performed a number of high-throughput screens in which the donor *pigP* elements were mobilised using a genomic Jumpstarter stock expressing the *piggyBac* transposase. The progeny embryos from these mobilisations were screened for YFP expression using a COPAS Select Embryo sorter (Fig. 1C,D) (Buszczak et al., 2007). For each *pigP* vector, we used a number of different donor lines carried on marked balancer chromosomes or on the 4th chromosome to counteract any donor-specific mobilisation bias. The vast majority of mobilisations were carried out in the female germline to increase the representation of protein traps on the X chromosome and so that we could detect inserts in maternally expressed proteins that perdure into the embryo. We used a total of 60 different starting lines and screened over 41 million embryos, yielding over 12,000 positive single embryos (0.03%) that gave rise to 4504 adults of which 1092 were confirmed as YFP positive after rescreening (supplementary material Table S1). Each line was given a CPTI designation (Cambridge Protein Trap Insertion) and the site of the insertion was mapped to the *Drosophila* genome sequence by inverse PCR (Liao et al., 2000).

After balancing and discarding multiple identical or very similar insertions, we retained a total of 616 CPTI lines, of which 604 were unambiguously mapped to genomic locations (Fig. 1E; supplementary material Table S2) and 16 lines were verified by 5′ or 3′ RACE (supplementary material Table S3). Eight of the 12 lines that could not be mapped by sequencing show YFP expression patterns consistent with protein traps and five are lethal or semi-lethal. We have not analysed these lines further but they may represent new genes or new exons of known genes. Five hundred and twenty six (85%) of the lines carried insertions into introns between protein coding exons in the orientation and reading frame expected for bona fide protein traps, generating protein traps in 346 unique genes. The remaining 79 lines could either not be mapped because they were insertions into repetitive sequence or were intronic insertions in the wrong frame (13 lines), the wrong orientation (six lines) or within an intron annotated as a UTR (nine lines). We examined these in more detail and showed that four lines have 5′ or 3′ RACE data that support the proposed protein trap insertions (bold in supplementary material Table S3). We next examined the interaction and YFP expression data described below for evidence supporting proposed protein traps. For four lines we have mass spectrometry data identifying peptides from the proposed protein trap. For 45 lines, the YFP expression annotations and sub-cellular localisations are consistent with protein traps in the proposed genes, and this evidence is particularly compelling for 31 of these. We have therefore included these unverified lines in the list of protein traps, giving a total of 575 lines trapping 374 unique genes. The supporting evidence for each protein trap line is indicated in supplementary material Table S2.

Of the unique genes we trapped, 228 have associated lethal alleles in FlyBase. We have phenotypic information on our protein trap insertions in 223 of these genes, of which 148 (66%) are homozygous viable, including insertions in haplo-insufficient genes such as *Notch* and *Ubx*. For 63 (28%) of the genes, we recovered only lethal insertions and the remaining 12 (5%) contained semi-lethal or sterile insertions. Thus, more than two-thirds of protein trap insertions in essential genes yield at least partially functional proteins. Some significant examples include homozygous viable insertions in *α-Catenin*, *armadillo*, *CaMKII*, *emc*, *Notch*, *Ubx* and *zipper*. Although we cannot directly assess the proportion of inserts in non-essential genes that are functional, this is likely to be similar to that of essential genes.

Overall, we have strong evidence for protein trap insertions in 374 annotated genes and we compared this list with the verified protein traps reported in the FlyTrap database (Morin et al., 2001; Buszczak et al., 2007; Quinones-Coello et al., 2007). Analysing the lists of trapped genes in FlyMine to account for any annotation differences, we identified a combined total of 514 trapped genes, of which 115 (22%) are common to both screens, 146 (28%) are unique to the Carnegie collection and 263 (51%) are unique to our new collection (supplementary material Table S4). Thus, we have doubled the number of *Drosophila* proteins that have been tagged using this approach. We compared the general properties of the trapped genes with the entire genome and found that trapped loci have significantly more introns than the genome average (mean intron number 5.77 versus 2.35, *P*<1E–16) and the average size of trapped introns is significantly larger than the genome average (mean intron size 8900 bp versus 826, *P*<1E–16). As expected, many of the trapped proteins are widely expressed during embryonic development according to BDGP expression pattern annotations. Looking at functional categories associated with the 387 trapped genes (supplementary material Table S5), we observed a significant over-representation of proteins annotated in cellular junction (4.8E–10), fusome (4.6E–08) and cytoskeletal (*P*=8.8E–07) components, which is reflected in over-representation of cytoskeletal protein binding (2.0E–04) as an annotated molecular function. Over 56% (213) of the trapped genes have GO annotations associated with development (*P*=1.9E–25), with a highly significant over-representation of genes involved in specific processes such as cytoskeletal organisation (1.3E–16), nervous system development (1.5E–15) and oogenesis (1.0E–11). These observations correlate well with the expression annotations we describe below.

### Expression patterns and subcellular localisations of the trapped proteins

To characterise the CPTI lines, we assembled a consortium of 22 different research groups in the UK who screened the lines in a variety of tissues and organs at various stages of development. Each screening group then deposited representative images showing the distribution of each protein trap line in their tissue of interest into the Flannotator database (http://www.flyprot.org/), along with an annotation using a controlled vocabulary that describes the expression pattern and subcellular distribution of the trapped protein in the tissue (Ryder et al., 2009). The screening groups deposited nearly 7000 annotations into the database, which allowed us to automatically compile a summary of the tissues in which each protein is expressed throughout development and to produce a key word cloud that can be used to identify lines with similar patterns.

**Fig. 2.**
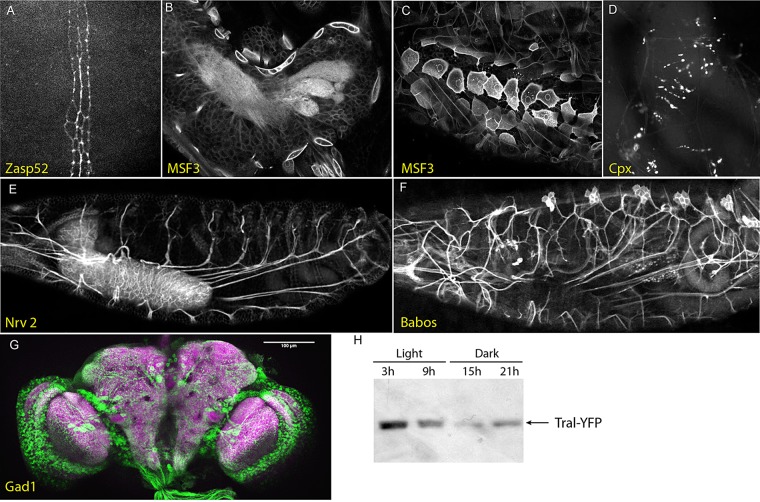
**Examples of protein trap lines with tissue-specific expression patterns.** (A) Zasp52 (CPTI-000408) is expressed in two rows of ventral mesectoderm cells in the germ band extending embryo. (B,C) The putative glutamate transporter MSF3 (CPTI-002305) is expressed in the first instar larva in protrusive cells that envelop the central nervous system, which are likely to be the surface glia that form the blood-brain barrier. (D) The CPTI-001473 insertion in Complexin labels the neuromuscular junctions in the first instar larva. (E) An insert in Nervana 2 (CPTI-001459), the β subunit of the Na^+^/K^+^ ATPase, is strongly expressed in the central nervous system and labels the axons of the motor neurons extending to their target muscles. (F) A Babos protein trap insertion (CPTI-0001423) labels the peripheral nervous system, including the sensory axons projecting towards the CNS. (G) A maximum intensity projection of a *z*-stack through the adult brain showing the expression pattern of Gad1 (CPTI-000977; green) and stained for Bruchpilot (magenta). (H) A western blot probed with mouse monoclonal anti-GFP, showing the circadian expression of the CPTI-100059 insert in Trailer hitch (Tral) in extracts from adult heads. Flies were grown at 18°C under a 12 h light/12 h dark regime with samples taken at the times indicated.

By screening the CTPI lines at multiple stages of development, we were able to identify many expression patterns that provide useful markers for specific structures and suggest new functions for the trapped proteins. For example, the embryo screens revealed that the PDZ- and LIM-domain protein Zasp52 (CPTI-000408) is specifically expressed in two lines of mesectoderm cells along the ventral midline, suggesting that this integrin regulator may play a specific role in these cells (Bouaouina et al., 2012) (Fig. 2A). MSF3 (CPTI-002305) is highly expressed in the first larval instar in the plasma membranes of protrusive cells around the central nervous system that are probably the surface glia that form the blood-brain barrier (Schwabe et al., 2005) (Fig. 2B,C). This Na^+^-dependent inorganic phosphate co-transporter is an orthologue of mammalian glutamate transporters, and may play a role in regulating glutamate levels in the central nervous system. The first instar larval screen identified several other insertions that provide useful markers for specific neural structures. Among these are: an insert in Complexin (CPTI-001473) that specifically marks neuromuscular junctions, consistent with the role of this protein in synaptic vesicle release (Jorquera et al., 2012); an insert in the Na^+^/K^+^ ATPase β-subunit Nervana 2 (CPTI-001459) that strongly labels axonal membranes and highlights the paths of the motor axons from the CNS to their target muscles; and an insert in Babos (CPTI-0001423) that labels the peripheral nervous system (Fig. 2D-F). The utility of protein trap lines as markers for regions of the nervous system is further highlighted by the screen for patterns in the adult brain, such as that shown by Gad1 (CPTI-000977) (Fig. 2G). Because of the complex three-dimensional structure of the brain, serial optical sections are necessary to interpret the protein trap expression patterns properly, and 535 of these are available as stacks in the Braintrap database (http://fruitfly.inf.ed.ac.uk/braintrap/) (Knowles-Barley et al., 2010). Not all of the screens were for spatial expression patterns and the Leicester group used western blots to identify proteins whose levels fluctuate with a circadian rhythm during a normal light/dark cycle, such as Trailer hitch (CPTI-1000059) (Fig. 2H).

**Fig. 3.**
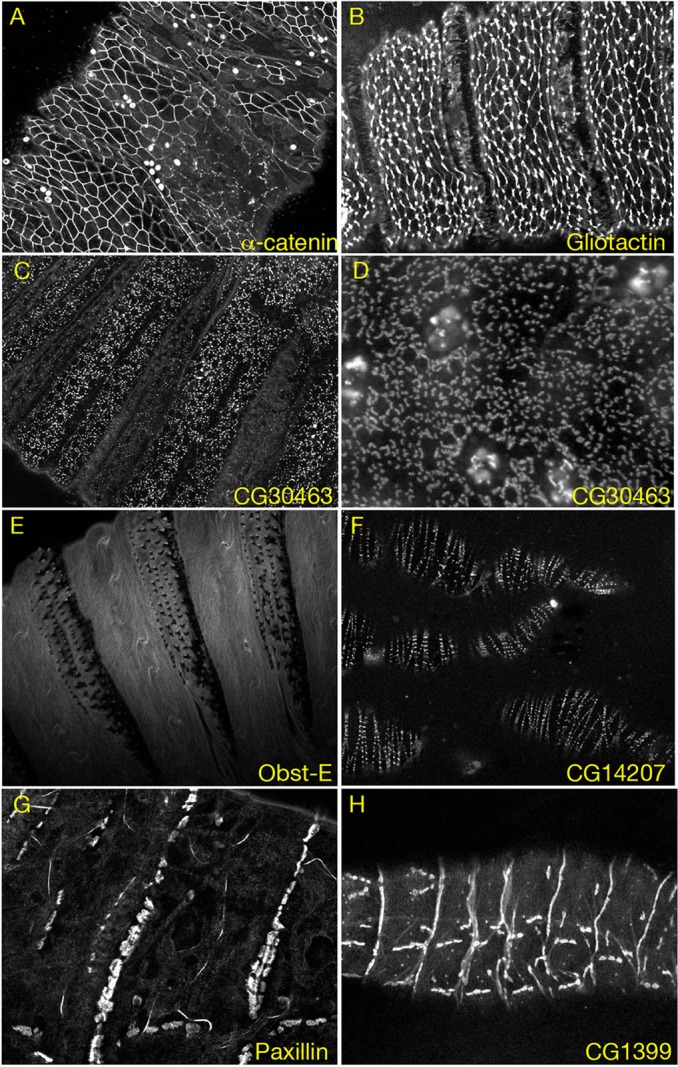
**Examples of protein trap insertions with specific subcellular localisations.** (A) CPTI-002342 (α-catenin) labels the adherens junctions that outline the apical margins of the cells of the larval epidermis. (B) Gliotactin (CPTI-003903) strongly accumulates at the tricellular junctions at the apical vortices of the larval epidermal cells. (C) The N-acetylgalactosaminyltransferase CG30463 (CPTI-003680) labels the dispersed Golgi ministacks in the larval epidermis. (D) CG30463 (CPTI-002151) localisation in the cells of the accessory gland of the adult male testis. (E) The CPTI-100048 insertion in the chitin-binding protein Obst-E marks whorls of chitin fibres in the first instar larval cuticle. (F) The CPTI-004445 insertion in CG14207 labels the *z* bands of the muscles in the ovarian sheath. (G,H) Paxillin (CPTI-000546) and the leucine-rich repeat protein CG1399 (CPTI-001765) are highly enriched at muscle-attachment sites in the first instar larva.

Perhaps the most valuable feature of protein trap screens is their ability to provide markers for subcellular structures and reveal previously unknown features of cellular organisation. The subcellular localisations of the protein trap lines in the early embryo are characterised in detail in the accompanying paper (Lye et al., 2014). Here, we focus on subcellular patterns that are most apparent at other stages of development using the primary data from the Flannotator database. Several markers may prove useful for tracking morphogenesis in epithelial tissues, including a viable insert in α-catenin (CPTI-002342) that provides a good marker for engaged cadherin at the adherens junctions, and a viable insert in Gliotactin (CPTI-003903) that highlights the tricellular junctions at cell vertices (Fig. 3A,B). Another potentially valuable class of inserts are those that label specific organelles, such as the two inserts in the N-acetylgalactosaminyltransferase CG30463 (CPTI-002151 and CPTI-003680) that strongly label the dispersed Golgi stacks of the larval epidermis (Fig. 3C). The Golgi forms longer ribbons in the testis accessory gland, revealing that the CG30463 protein localises to a single cisterna per stack (Fig. 3D). Other patterns can be detected only in a single tissue. For example, an insert in the chitin-binding protein Obst-E (CPTI-100048) labels the intricate pattern of chitin fibres in the larval cuticle, with whorls around sensory structures in regions of naked cuticle (Fig. 3E).

Three of the trapped proteins localise to the Z-lines of the muscles, two of which are novel (CPTI-002762; CG1674 and CPTI-004445; CG14207) (Fig. 3F). Similarly, 18 proteins are enriched at muscle-attachment sites: these include several well-characterised components of these attachments, such as Pax (CPTI-000546) and Shortstop (Shot; CPTI-001962) (Strumpf and Volk, 1998; Brown et al., 2000), but most had not been previously identified, including several proteins that are highly specific for this site, such as the leucine-rich repeat protein CG1399 (CPTI-001765) (Fig. 3G,H).

**Fig. 4.**
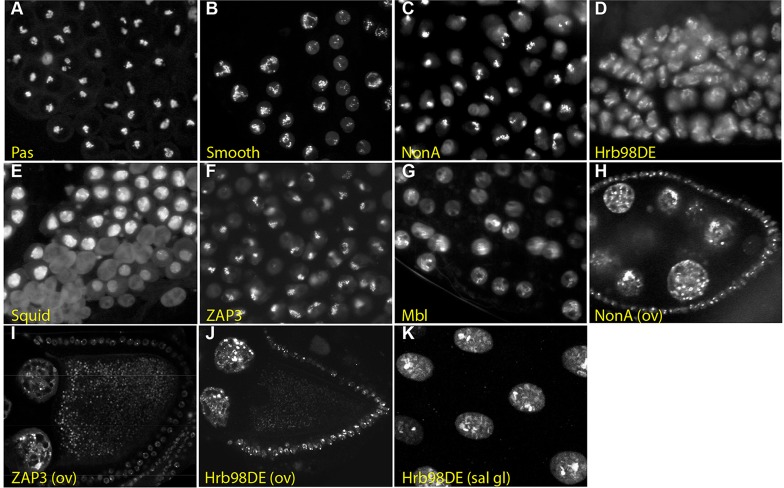
**Protein trap lines that label intranuclear structures in primary spermatocytes that are likely to correspond to the giant loops of the Y chromosome.** (A) Pasilla (CPTI-000668) marks the C-loop of the Y chromosome in primary spermatocytes. (B-F) Smooth (CPTI-002828) (B), NonA (CPTI-003091) (C), Hrb98DE (CPTI-000205) (D), Squid (CPTI-000239) (E), ZAP3 (CPTI-004292) (F) and Muscleblind (CPTI-003555) (G) label similar structures that are likely to be giant loops of the Y chromosome. (H-J) NonA (H), ZAP3 (I) and Hrb98DE (J) also mark intranuclear speckles in the nurse cells and follicle cells of the ovary. (K) Hrb98DE localisation on the polytene chromosomes of the larval salivary gland.

The screens of the testes and ovaries identified a number of proteins that localise to structures that are unique to either the male or female germ line. One striking example in the testis is the localisation of the multi-KH domain, RNA-binding protein Pasilla (CPTI-000668, CPTI-001063 and CPTI-001261) to a thread-like intranuclear structure in primary spermatocytes (Fig. 4A). This structure presumably corresponds to the C-loop of the Y chromosome, which is a large lampbrush-like chromosomal loop that is transcribed to produce a primary transcript of over 1 Mb (Redhouse et al., 2011). Interestingly, several other RNA-binding proteins show similar localisations in primary spermatocytes. These proteins include: the hnRNP A, A/B and L orthologues Hrb98DE (CPTI-000165, CPTI-000205 and CPTI-003669), Squid (CPTI-000239) and Smooth (CPTI-002653 and CPTI-002828), respectively; the alternative splicing regulators Muscleblind (CPTI-003555) and NonA (CPTI-003091); and the putative protein phosphatase 1 regulator ZAP3 (CPTI-004292) (Fig. 4B-G). Thus, these proteins may also associate with specific regions of some of the large primary transcripts that are expressed from the Y chromosome loops. Most of these proteins also label intranuclear structures in other cell types. For example, ZAP3, NonA and Hrb98DE mark puncta in the nuclei of the nurse cells and follicle cells of the ovary, and Hrb98DE and Squid mark specific polytene bands in the salivary glands (Fig. 4H-K).

**Fig. 5.**
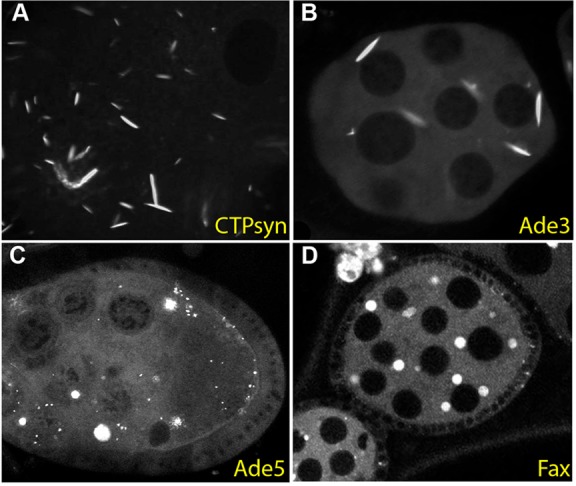
**Protein trap lines that label large cytoplasmic aggregates in the female germ line.** (A) A protein trap insertion in Cytidine synthase (CPTI-001881) labels the rod-like cytoophidia that form in the cytoplasm of the germ cells of the developing egg chamber. (B) Ade3 (CPTI-003733) forms similar rod-like structures in the female germ line. (C) Ade5 (CPTI-002207) forms more spherical aggregates in the nurse cell and oocyte cytoplasm. (D) Fax (CPTI-002774) also localises to large spherical cytoplasmic structures in these cells.

Previous protein trap screens identified a new class of subcellular structure in the female germ line called a cytoophidium, which is a large intracellular rod formed by aggregation of the enzyme Cytidine synthase (Liu, 2010; Noree et al., 2010). Our screen also isolated a protein trap in Cytidine synthase (CPTI-001881) that forms cytoophidia in the developing oocyte (Fig. 5A). In addition, we recovered three other lines that form large cytoplasmic structures in the female germ line. The first of these is Ade3 (CPTI-003733), which encodes the trifunctional enzyme – phosphoribosylglycinamide formyltransferase/phosphoribosylglycinamide synthetase/phosphoribosylaminoimidazole synthetase (GART) – that catalyses several steps in the purine biosynthesis pathway. GART has been observed to form filaments in purine-deprived human cells and in yeast cells in stationary phase, and forms similar rod-like filaments to CTP synthase in the female germ line (Fig. 5B) (An et al., 2008; Narayanaswamy et al., 2009). The next enzyme in this pathway, Ade5 [CPTI-002207; the bifunctional phosphoribosylaminoimidazole carboxylase/phosphoribosylaminoimidazole succinocarboxamide synthetase (PAICS)], also forms cytoplasmic aggregates in the female germ line, but these have a different shape from the GART and CTP synthase rods (Fig. 5C). Thus, enzymes necessary for both pyrimidine and purine biosynthesis are packaged into at least two types of large intracellular aggregate in the developing egg. As the formation of cytoophidia is enhanced by starvation or drugs that inhibit nucleotide production, these aggregates may act to increase the catalytic activity of these enzymes (Chen et al., 2011). The formation of these structures could therefore play an important role in producing the large quantities of nucleotides required for DNA and RNA synthesis in the oocyte to support the endoreduplication of the nurse cells and the production of very large numbers of ribosomes. The final protein that forms aggregates in the germ line is Failed axon connections (Fax; CPTI-002774), which encodes a protein of unknown function with a glutathione-S-transferase domain (Fig. 5D).

### Identification of *in vivo* interaction partners

The final component of the screen was to determine which proteins interact with the trapped proteins *in vivo* by affinity purifying the trapped proteins from embryonic extracts using the StrepTagII, 3×FLAG and YFP tags and identifying co-purifying proteins by mass spectrometry. The original large-scale proteomic screens for interactors in yeast used tandem affinity purification, in which the bait protein is affinity purified using one tag and re-purified using the second, as this yields cleaner purifications with fewer false positives (Gavin et al., 2002; Ho et al., 2002). However, this approach produced low yields when applied to *Drosophila* embryonic extracts, presumably because many of the trapped proteins are expressed only in a subset of embryonic cells at specific stages of development (Rees et al., 2011). We therefore performed parallel affinity purifications with the StrepTagII and either the 3×FLAG tag or YFP. A small aliquot of each affinity purification was first run on an SDS-PAGE gel, western blotted and probed for GFP, and only samples in which the bait was detectable were subjected to mass spectrometry. We carried out affinity purifications on 235 lines containing both the StrepTagII and 3×FLAG tags, and pulled down the trapped proteins from 205 lines. We performed an additional 28 parallel affinity purifications using StrepTagII and YFP, 26 of which were successful. Each pull-down was performed at least twice to assess the reproducibility of the protein interactions.

One-step affinity purifications suffer from a number of common contaminants; we took two approaches to deal with this issue. First, we employed an exclusion list in our mass-spectrometric experiments, so that ions corresponding to the most abundant peptides from ubiquitous, non-specific interacting proteins, such as actin and yolk proteins, were not sampled, as this was found to improve the detection of low-abundance bait proteins (Rees et al., 2011). These exclusion lists also included the most abundant peptides from proteins that bind to each affinity resin in the absence of a tagged bait protein, the so-called ‘BEADome’, ‘FLAGome’ and ‘STREPome’. However, we were reluctant to exclude too many peptides from our analyses, as this might prevent the detection of other peptides with the same m/z values. In addition, given the wide variety of trapped protein baits, it seemed likely that contaminants in some pull-downs might be real interactors in others. We therefore marked the other common contaminants in our lists of interacting proteins in Flannotator, so that users can choose whether or not to ignore these data. We also noticed that the pull-downs with protein trap lines, but not other types of tagged protein, often included some ribosomal proteins and protein-folding factors. This is probably due to a delay in protein folding caused by the insertion of the YFP cassette into the middle of the trapped proteins, which may prolong their retention at the ribosome exit site (Pechmann et al., 2013). We therefore also marked these as possible contaminants in our interaction lists, as they are less likely to represent bona fide interactions of the untagged native proteins. Finally, we marked all additional proteins that appeared in more than 10% of the pull-downs as putative contaminants.

**Fig. 6.**
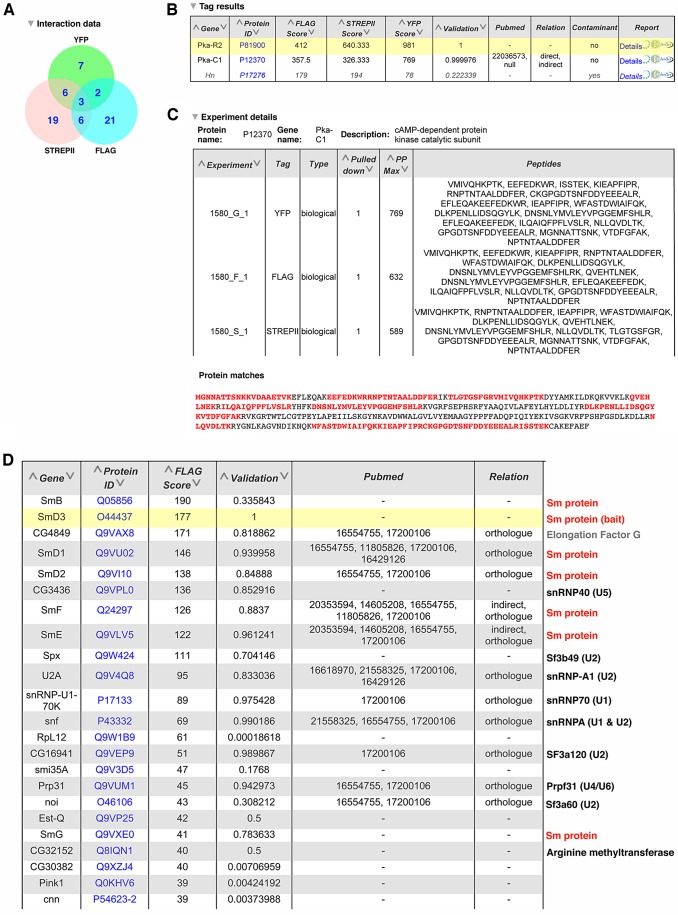
**Annotation of the protein interaction data from the affinity purifications of protein trap lines.** (A) A Venn diagram showing the number of proteins that co-purified with PKA-R2 (CPTI-001580) in the affinity purifications using the 3×FLAG, StrepTagII and YFP tags. (B) A section of a table generated in Flannotator listing the three proteins that were detected in all three purifications of PKA-R2. The bait protein is highlighted in yellow and likely contaminants are shown in grey. (C) The details of PKA-C1 peptides identified by the mass spectrometer in some of the 3×FLAG, StrepTagII and YFP affinity purifications of PKA-R2 (taken from Flannotator). The ‘Protein Matches’ section at the bottom shows the amino acid sequence of PKA-C1 with the positions of identified peptides highlighted in red. (D) A section of a table generated in Flannotator listing the proteins that co-purified with SmD3 in the 3×FLAG affinity purifications. These include the other six Sm proteins that form a heptameric ring with SmD3 (CPTI-002164) and components of the U1, U2, U4, U5 and U6 snRNPs, with which the Sm proteins associate. The ‘Validation’ column indicates the probability score that the observed interaction is real calculated using a Generalized Iterative Scaling-Maximum Entropy supervised machine-learning approach. The ‘Pubmed’ column lists the PubMed identification numbers of any publications that have also described an interaction between SmD3 and the identified protein.

All of these affinity purification data were submitted to the IMEx consortium (http://www.imexconsortium.org) through IntAct (Orchard et al., 2014) and are summarised in the Interactions section of the Flannotator record for each line (marked by a circumflex in the browser view). An example of the proteomics data is shown in Fig. 6A for an insertion PKA-R2. The top level shows a Venn diagram displaying the number of proteins identified in the 3×FLAG, StrepTagII and YFP affinity purifications, as well as the proteins in each pull-down that were present in pull-downs with another tag (Fig. 6A). Fig. 6B shows part of a table generated in Flannotator containing the identity of the proteins ranked by the MASCOT protein probability score with the bait protein highlighted in yellow and the likely contaminants listed at the end. For example, PKA-C1 was the only protein that co-purified with PKA-R2 in the 3×FLAG, StrepTagII and YFP pull-downs that was not a common contaminant. The proteins that co-purified with each single tag or combination of tags can be displayed by clicking on the appropriate entry in the Venn diagram. In Fig. 6C (generated in Flannotator), the ‘Details’ section for each interactor shows the peptides from the protein identified by the mass spectrometer, along with their positions in the protein sequence, as shown for PKA-C2.

As with all large-scale interaction screens, our interaction lists are still likely to contain a proportion of false positives, even after removing the common contaminants. We therefore took two strategies to estimate the confidence of each interaction. First, we used a supervised machine-learning approach to evaluate the likelihood that a given interaction was real, using a positive training set based on the *Drosophila* orthologues of curated protein complexes from *S. cerevisiae* (Pu et al., 2009). This assigned scores between 0 and 1 to each protein interaction, with 0 representing the lowest probability that the two proteins associate *in vivo* and 1 the highest. Second, we used FlyMine to determine whether any of the interactions had been also observed in other publically available datasets, as interactions that have been detected in multiple experiments are much more likely to be bona fide, especially when these use complementary approaches, such as affinity purifications and yeast two-hybrid screens (von Mering et al., 2002; Schwartz et al., 2009). We identified matches in databases of putative direct interactions (yeast two-hybrid) and indirect interactions (affinity purifications, yeast two-hybrid interactions with one intermediary protein and genetic interactions) in *Drosophila*, as well as interactions between the orthologous proteins in other species. These high-confidence interactions are indicated in the relation column of each table in Flannotator, along with the PubMed IDs of the relevant publications (Fig. 6D).

A comparison with the tandem affinity purification of tagged proteins from *Drosophila* tissue culture cells (Guruharsha et al., 2011) reveals that the 49 bait proteins common to both screens identified 318 of the same interactors, but two-thirds of these (202) were classified as likely contaminants according to our criteria. Some of this latter class may be bona fide interactors, but the majority are probably sticky proteins that bind non-specifically to affinity purification matrices. A lower proportion of the interactions that were also observed in yeast two-hybrid screens fall into the possible contaminant class, with 231/444 classed as high-confidence interactors (Giot et al., 2003; Formstecher et al., 2005). This illustrates the advantage of verifying protein:protein interactions by comparing different types of interaction data that are less likely to share the same false positives.

After removing 6714 likely contaminants, the affinity purifications identified 14,932 putative protein-protein interactions, of which 426 are high-confidence interactions supported by other data. These include a number of well-characterized protein complexes, such as the proteasome (CPTI-002234), the V-ATPase (CPTI-002280 and CPTI-100041), the Ino80 chromatin modifying complex (CPTI-001224), the myosin phosphatase complex (CPTI-001360) and the H/ACA ribonucleoprotein complex (CPTI-002287), indicating that protein complexes were effectively purified from multiple sub-cellular compartments. Fig. 6D shows the proteins with the highest confidence scores (selected from Flannotator) that co-purified with SmD3 (CPTI-002164), one of the seven Sm proteins that form a heptameric ring associated with the U1, U2, U4 and U5 small uridine-rich RNAs of the spliceosome (Will and Luhrmann, 2001; Herold et al., 2009). All of the other six Sm proteins co-purify with SmD3, as well as subunits of the U1, U2, U4/U6 and U5 snRNPs. Several of the Sm proteins undergo symmetric arginine dimethylation, which is thought to be catalysed by the Dart5 arginine methyltransferase (Gonsalvez et al., 2006). Mutations in *dart5* do not disrupt splicing, however, suggesting that other arginine methylases might also play a role. It is therefore interesting that the uncharacterised arginine methylase, CG32152, also co-purifies with SmD3.

## DISCUSSION

Here, we report the generation of a protein trap library using a hybrid *piggyBac*/*P* element vector and the characterisation of the expression patterns, subcellular localisations and *in vivo* interaction partners of the resulting protein trap insertions. Our screen identified 616 new insertions in 374 genes of which 263 are novel, and we have therefore significantly increased the number of *Drosophila* genes that have been tagged with protein trap lines insertions. All of the lines have been deposited in the *Drosophila* Genetic Resource Center at the Kyoto Institute of Technology (http://www.dgrc.kit.ac.jp/), and are available for use by the community.

The *pigP* protein trap library provides a versatile resource for studying the behaviour and function of proteins *in vivo*, as shown by the more than 20 publications that have already reported results using insertions in this library (Monier et al., 2009; Knowles-Barley et al., 2010; Choo et al., 2011; Hijazi et al., 2011; Peng et al., 2011; Redhouse et al., 2011; Syed et al., 2011; Fischer et al., 2012; Neumuller et al., 2012; O'Sullivan et al., 2012; Olesnicky et al., 2012; Timofeev et al., 2012; Zhao et al., 2012; Huelsmann et al., 2013; Lewellyn et al., 2013; Manhire-Heath et al., 2013; Marinho et al., 2013; Morais-de-Sa et al., 2013; Schneider et al., 2013; Yamamoto et al., 2013). One advantage of protein trap insertions is that they are expressed at endogenous levels under the control of their native regulatory elements, which makes them excellent markers for protein localisation *in vivo*. This contrasts with Gal4/UAS reporter constructs, which are usually overexpressed compared with the endogenous protein, and many genomic transgenes, which are often expressed at different levels depending on the genomic context of their insertion sites. Although our protein trap lines are tagged with YFP, which limits their use to one line at a time, we note that the *P* element ends within the *pigP* element facilitate straightforward exchange of the fluorescent protein tag by *P* element exchange (Gloor et al., 1991; Sepp and Auld, 1999). We have generated *pigP* transgenic lines with the red fluorescent protein Cherry in place of YFP, and have used these successfully to convert several protein traps from yellow to red fluorescence.

A second advantage of protein trap lines is that they provide several ways to examine the loss of function phenotypes of the trapped genes. First, one can generate imprecise excisions of the *pigP* elements by providing a source of *P* transposase to mobilise the *P* element ends and screening for imprecise excisions (Adams and Sekelsky, 2002). In many cases, however, it is more convenient to knock down gene function in a specific tissue or at a precise stage of development. This can be achieved by using UAS-driven shRNAs that efficiently target GFP and YFP (Neumuller et al., 2012). Targeting a YFP protein trap rather than the endogenous mRNA is advantageous as the shRNAs targeting YFP are known not to cause off target effects and the effectiveness of the RNAi can be monitored by measuring the loss of YFP signal. The time taken for RNAi to knock down gene function depends on the half-life of the protein and there is an inevitable delay before the residual protein decays. This problem has been elegantly overcome by the development of the deGradFP technique, in which the trapped protein is targeted for degradation directly by an anti-GFP/YFP nanobody fused to the F-box of the SCF-Slimb Ubiquitin ligase (Caussinus et al., 2012). This approach makes it possible to degrade the tagged protein rapidly upon induction of the nanobody fusion. Another option for ablating protein function that provides even more temporal and spatial control is chromophore-assisted laser inactivation, which uses a focused laser beam to inactivate the YFP-containing protein very rapidly at a specific subcellular location (Monier et al., 2010).

The protein traps in our library differ from previous protein trap collections by the inclusion of two or more protein affinity tags within the artificial exon, facilitating purification of the trapped proteins and identification of co-purifying factors via mass spectrometry. Because our aim was to analyse samples in a high-throughput fashion, our protocols were optimised for processing large numbers of lines in parallel (Rees et al., 2011). This worked well for many lines, generating a large amount of new protein-protein interaction data that confirm many low-confidence interactions that had previously been observed only in yeast two-hybrid screens. Some trapped proteins were not purified under these conditions, however, or they pulled down only contaminating proteins. This problem might be addressed by optimising the protocol for the individual proteins, or by performing tandem-affinity purifications on isolated tissues or cells in which the proteins of interest are most highly expressed.

One of the goals of our screen was to recover protein trap insertions in genes that are refractory to *P* element insertions by using a *piggyBac* vector that should insert more randomly in the genome (Thibault et al., 2004; Bellen et al., 2011). This approach was partially successful, in that we recovered many insertions in genes that had not been targeted in the *P* element protein trap screens, which doubled the number of tagged genes overall. However, this only represents just over 4% of the potentially ‘trappable’ genes with introns that are expressed in the embryo (Graveley et al., 2011). Nevertheless, it seems likely that the screen came close to saturating the proteins that can be trapped by the *pigP* protein trap vector, as 158 of the 387 genes with insertions were hit more than once. This tendency to insert in the same genes multiple times is unlikely to be due to local hotspots of *pigP* insertion, because several of inserts in the same gene are over 50 kb apart, with the inserts in *Ten-m* being separated by 85 kb. Indeed, this proved useful in some cases, as 11 out the 44 genes with inserts in different introns have both viable and lethal/semi-lethal insertions.

The apparent near saturation of our *pigP* protein trap screen could be due in part to a bias against *piggyBac* insertions in regions of the genome that have a specific chromatin state. Screens based on a different transposon, such as *Minos*, which inserts somewhat more randomly than *piggyBac*, might therefore improve the coverage of trapped proteins (Bellen et al., 2011). However, insertional bias is probably only a minor factor in explaining why only a small proportion of the proteome has been trapped to date. Only 25% of the genes that are hotspots for *piggyBac* insertion were identified in our screens, but all but one of hotspot genes that were hit had multiple insertions. This suggests that most other hotspot loci do not produce detectable YFP-tagged proteins in the embryo when the *pigP* vector is inserted. To be identified in a protein trap screen, the tagged protein must form a stable product with YFP inserted internally and it must also be expressed at sufficient levels by enough cells in the embryo to be detectable with the COPAS embryo sorter. Because proteins are unstable if misfolded, and many proteins are expressed only in specific cell types at particular stages of development, many proteins are probably refractory to detection in protein trap screens. A previous analysis suggested that successful protein traps are most likely to be recovered when the insertion occurs in a disordered or surface-exposed region of the protein, and this may also contribute to our apparent near saturation in the screen (Aleksic et al., 2009). More complete coverage of the proteome will therefore require reverse genetic approaches, such as using recombination-mediated cassette exchange to place fluorescent tags into MiMic insertions in appropriate introns or recombineering of P[acMan] or FlyFOS clones to target the fluorescent tags to positions that do not affect protein folding (Venken et al., 2006, 2011; Ejsmont et al., 2009). Although such reverse genetic approaches are more labour intensive, they have the advantage that one can focus on the specific tissues where the protein is most highly expressed, which should improve the detection of low-abundance proteins.

## MATERIALS AND METHODS

### Protein trap vectors

Synthetic exons were based on the constructs used in the original *Drosophila* protein trap screen using splice acceptor and donor sequences from the *Drosophila Mhc* gene (Morin et al., 2001). The original GFP sequences were replaced with Venus YFP flanked by affinity tags to allow for purification of tagged proteins. The nested protein trap *pigP* constructs were made by inserting *P* element-based protein trapping sequences into a unique *Hpa*I site in the *piggyBac* vector p3E1.2, which has an intact *piggyBac* element (Fraser et al., 1995). Details of the vectors used are provided in supplementary Materials and Methods, with graphical representations presented in supplementary material Fig. S1. Complete sequence and maps of the constructs used are available at http://www.flyprot.org/construct_notes.php.

### Fly stocks and screens

*Drosophila* stocks were maintained at 25°C on standard cornmeal agar. *piggyBac* mobilisations were performed as exemplified in the crossing schemes described in supplementary Materials and Methods using J10 or J6 pMos{3×P3-ECFP, αtub-*piggyBac*K10} transposase sources (Horn et al., 2003). Virgin collection was simplified by using *P{hs-hid}Y* to eliminate males (FlyBase). Embryos from dysgenic crosses were collected on freshly yeasted apple-juice agar plates and individual YFP-positive embryos were selected using the COPAS Select (Union Biometrica). Single embryos were collected in 24-well apple juice agar plates, surviving L3 larvae were transferred to individual yeasted cornmeal agar tubes and eclosing adults were crossed as described in supplementary Materials and Methods. Embryos were collected from established lines and YFP expression confirmed by sorting with the COPAS Select. Positive lines were mapped to the *Drosophila* genome via inverse-PCR or 5′ and 3′ RACE (Liao et al., 2000). Manipulation of gene lists and assessment of gene ontology enrichments (Holm-Bonferroni corrected for multiple testing and corrected for gene length) were performed in FlyMine (Lyne et al., 2007).

### Affinity purifications

Affinity purifications were performed as described by Rees et al. (2011) and are described in detail in the supplementary Materials and Methods.

## Supplementary Material

Supplementary Material

## References

[DEV111054C1] Adams, M. D. and Sekelsky, J. J. (2002). From sequence to phenotype: reverse genetics in Drosophila melanogaster. *Nat. Rev. Genet.* 3, 189-198 10.1038/nrg752 11972156

[DEV111054C2] Adams, M. D., Celniker, S. E., Holt, R. A., Evans, C. A., Gocayne, J. D., Amanatides, P. G., Scherer, S. E., Li, P. W., Hoskins, R. A., Galle, R. F. et al. (2000). The genome sequence of Drosophila melanogaster. *Science* 287, 2185-2195 10.1126/science.287.5461.2185 10731132

[DEV111054C3] Aebersold, R. and Mann, M. (2003). Mass spectrometry-based proteomics. *Nature* 422, 198-207 10.1038/nature01511 12634793

[DEV111054C4] Aleksic, J., Lazic, R., Müller, I., Russell, S. R. and Adryan, B. (2009). Biases in Drosophila melanogaster protein trap screens. *BMC Genomics* 10, 249 10.1186/1471-2164-10-249 19476619 PMC2695487

[DEV111054C5] An, S., Kumar, R., Sheets, E. D. and Benkovic, S. J. (2008). Reversible compartmentalization of de novo purine biosynthetic complexes in living cells. *Science* 320, 103-106 10.1126/science.1152241 18388293

[DEV111054C6] Arbeitman, M. N., Furlong, E. E. M., Imam, F., Johnson, E., Null, B. H., Baker, B. S., Krasnow, M. A., Scott, M. P., Davis, R. W. and White, K. P. (2002). Gene expression during the life cycle of Drosophila melanogaster. *Science* 297, 2270-2275 10.1126/science.1072152 12351791

[DEV111054C7] Bellen, H. J., Levis, R. W., Liao, G., He, Y., Carlson, J. W., Tsang, G., Evans-Holm, M., Hiesinger, P. R., Schulze, K. L., Rubin, G. M. et al. (2004). The BDGP gene disruption project: single transposon insertions associated with 40% of Drosophila genes. *Genetics* 167, 761-781 10.1534/genetics.104.026427 15238527 PMC1470905

[DEV111054C8] Bellen, H. J., Levis, R. W., He, Y., Carlson, J. W., Evans-Holm, M., Bae, E., Kim, J., Metaxakis, A., Savakis, C., Schulze, K. L. et al. (2011). The Drosophila gene disruption project: progress using transposons with distinctive site specificities. *Genetics* 188, 731-743 10.1534/genetics.111.126995 21515576 PMC3176542

[DEV111054C9] Bouaouina, M., Jani, K., Long, J. Y., Czerniecki, S., Morse, E. M., Ellis, S. J., Tanentzapf, G., Schock, F. and Calderwood, D. A. (2012). Zasp regulates integrin activation. *J. Cell Sci.* 125, 5647-5657 10.1242/jcs.103291 22992465 PMC3575701

[DEV111054C10] Brown, N. H., Gregory, S. L. and Martin-Bermudo, M. D. (2000). Integrins as mediators of morphogenesis in Drosophila. *Dev. Biol.* 223, 1-16 10.1006/dbio.2000.9711 10864456

[DEV111054C11] Buszczak, M., Paterno, S., Lighthouse, D., Bachman, J., Planck, J., Owen, S., Skora, A. D., Nystul, T. G., Ohlstein, B., Allen, A. et al. (2007). The carnegie protein trap library: a versatile tool for Drosophila developmental studies. *Genetics* 175, 1505-1531 10.1534/genetics.106.065961 17194782 PMC1840051

[DEV111054C12] Caussinus, E., Kanca, O. and Affolter, M. (2012). Fluorescent fusion protein knockout mediated by anti-GFP nanobody. *Nat. Struct. Mol. Biol.* 19, 117-121 10.1038/nsmb.2180 22157958

[DEV111054C13] Celniker, S. E., Wheeler, D. A., Kronmiller, B., Carlson, J. W., Halpern, A., Patel, S., Adams, M., Champe, M., Dugan, S. P., Frise, E. et al. (2002). Finishing a whole-genome shotgun: release 3 of the Drosophila melanogaster euchromatic genome sequence. *Genome Biol.* 3, RESEARCH0079 10.1186/gb-2002-3-12-research0079 12537568 PMC151181

[DEV111054C14] Chen, K., Zhang, J., Tastan, O. Y., Deussen, Z. A., Siswick, M. Y.-Y. and Liu, J.-L. (2011). Glutamine analogs promote cytoophidium assembly in human and Drosophila cells. *J. Genet. Genomics* 38, 391-402 10.1016/j.jgg.2011.08.004 21930098

[DEV111054C15] Choo, S. W., White, R. and Russell, S. (2011). Genome-wide analysis of the binding of the Hox protein Ultrabithorax and the Hox cofactor Homothorax in Drosophila. *PLoS ONE* 6, e14778 10.1371/journal.pone.0014778 21483667 PMC3071696

[DEV111054C16] Clyne, P. J., Brotman, J. S., Sweeney, S. T. and Davis, G. (2003). Green fluorescent protein tagging Drosophila proteins at their native genomic loci with small P elements. *Genetics* 165, 1433-1441. 14668392 10.1093/genetics/165.3.1433 PMC1462835

[DEV111054C17] Daniels, S. B., McCarron, M., Love, C. and Chovnick, A. (1985). Dysgenesis-induced instability of rosy locus transformation in Drosophila melanogaster: analysis of excision events and the selective recovery of control element deletions. *Genetics* 109, 95-117. 2981758 10.1093/genetics/109.1.95 PMC1202486

[DEV111054C18] Dietzl, G., Chen, D., Schnorrer, F., Su, K.-C., Barinova, Y., Fellner, M., Gasser, B., Kinsey, K., Oppel, S., Scheiblauer, S. et al. (2007). A genome-wide transgenic RNAi library for conditional gene inactivation in Drosophila. *Nature* 448, 151-156 10.1038/nature05954 17625558

[DEV111054C19] Ejsmont, R. K., Sarov, M., Winkler, S., Lipinski, K. A. and Tomancak, P. (2009). A toolkit for high-throughput, cross-species gene engineering in Drosophila. *Nat. Methods* 6, 435-437 10.1038/nmeth.1334 19465918

[DEV111054C20] Fischer, B. E., Wasbrough, E., Meadows, L. A., Randlet, O., Dorus, S., Karr, T. L. and Russell, S. (2012). Conserved properties of Drosophila and human spermatozoal mRNA repertoires. *Proc. Biol. Sci.* 279, 2636-2644 10.1098/rspb.2012.0153 22378807 PMC3350705

[DEV111054C21] Formstecher, E., Aresta, S., Collura, V., Hamburger, A., Meil, A., Trehin, A., Reverdy, C., Betin, V., Maire, S., Brun, C. et al. (2005). Protein interaction mapping: a Drosophila case study. *Genome Res.* 15, 376-384 10.1101/gr.2659105 15710747 PMC551564

[DEV111054C22] Fraser, M. J., Cary, L., Boonvisudhi, K. and Wang, H.-G. H. (1995). Assay for movement of Lepidopteran transposon IFP2 in insect cells using a baculovirus genome as a target DNA. *Virology* 211, 397-407 10.1006/viro.1995.1422 7645244

[DEV111054C23] Gavin, A.-C., Bosche, M., Krause, R., Grandi, P., Marzioch, M., Bauer, A., Schultz, J., Rick, J. M., Michon, A.-M., Cruciat, C.-M. et al. (2002). Functional organization of the yeast proteome by systematic analysis of protein complexes. *Nature* 415, 141-147 10.1038/415141a 11805826

[DEV111054C24] Giot, L., Bader, J. S., Brouwer, C., Chaudhuri, A., Kuang, B., Li, Y., Hao, Y. L., Ooi, C. E., Godwin, B., Vitols, E. et al. (2003). A protein interaction map of Drosophila melanogaster. *Science* 302, 1727-1736 10.1126/science.1090289 14605208

[DEV111054C25] Gloor, G. B., Nassif, N. A., Johnson-Schlitz, D. M., Preston, C. R. and Engels, W. R. (1991). Targeted gene replacement in Drosophila via P element-induced gap repair. *Science* 253, 1110-1117 10.1126/science.1653452 1653452

[DEV111054C26] Gonsalvez, G. B., Rajendra, T. K., Tian, L. and Matera, A. G. (2006). The Sm-protein methyltransferase, dart5, is essential for germ-cell specification and maintenance. *Curr. Biol.* 16, 1077-1089 10.1016/j.cub.2006.04.037 16753561

[DEV111054C27] Graveley, B. R., Brooks, A. N., Carlson, J. W., Duff, M. O., Landolin, J. M., Yang, L., Artieri, C. G., van Baren, M. J., Boley, N., Booth, B. W. et al. (2011). The developmental transcriptome of Drosophila melanogaster. *Nature* 471, 473-479 10.1038/nature09715 21179090 PMC3075879

[DEV111054C28] Guruharsha, K. G., Rual, J.-F., Zhai, B., Mintseris, J., Vaidya, P., Vaidya, N., Beekman, C., Wong, C., Rhee, D. Y., Cenaj, O. et al. (2011). A protein complex network of Drosophila melanogaster. *Cell* 147, 690-703 10.1016/j.cell.2011.08.047 22036573 PMC3319048

[DEV111054C29] Hacker, U., Nystedt, S., Barmchi, M. P., Horn, C. and Wimmer, E. A. (2003). piggyBac-based insertional mutagenesis in the presence of stably integrated P elements in Drosophila. *Proc. Natl. Acad. Sci. USA* 100, 7720-7725 10.1073/pnas.1230526100 12802016 PMC164654

[DEV111054C30] Herold, N., Will, C. L., Wolf, E., Kastner, B., Urlaub, H. and Luhrmann, R. (2009). Conservation of the protein composition and electron microscopy structure of Drosophila melanogaster and human spliceosomal complexes. *Mol. Cell. Biol.* 29, 281-301 10.1128/MCB.01415-08 18981222 PMC2612486

[DEV111054C31] Hijazi, A., Haenlin, M., Waltzer, L. and Roch, F. (2011). The Ly6 protein coiled is required for septate junction and blood brain barrier organisation in Drosophila. *PLoS ONE* 6, e17763 10.1371/journal.pone.0017763 21423573 PMC3058042

[DEV111054C32] Ho, Y., Gruhler, A., Heilbut, A., Bader, G. D., Moore, L., Adams, S.-L., Millar, A., Taylor, P., Bennett, K., Boutilier, K. et al. (2002). Systematic identification of protein complexes in Saccharomyces cerevisiae by mass spectrometry. *Nature* 415, 180-183 10.1038/415180a 11805837

[DEV111054C33] Horn, C., Offen, N., Nystedt, S., Hacker, U. and Wimmer, E. A. (2003). piggyBac-based insertional mutagenesis and enhancer detection as a tool for functional insect genomics. *Genetics* 163, 647-661. 12618403 10.1093/genetics/163.2.647 PMC1462455

[DEV111054C34] Huelsmann, S., Ylänne, J. and Brown, N. H. (2013). Filopodia-like actin cables position nuclei in association with perinuclear actin in Drosophila nurse cells. *Dev. Cell* 26, 604-615 10.1016/j.devcel.2013.08.014 24091012 PMC3791400

[DEV111054C35] Jorquera, R. A., Huntwork-Rodriguez, S., Akbergenova, Y., Cho, R. W. and Littleton, J. T. (2012). Complexin controls spontaneous and evoked neurotransmitter release by regulating the timing and properties of synaptotagmin activity. *J. Neurosci.* 32, 18234-18245 10.1523/JNEUROSCI.3212-12.2012 23238737 PMC3530744

[DEV111054C36] Knowles-Barley, S., Longair, M. and Armstrong, J. D. (2010). BrainTrap: a database of 3D protein expression patterns in the Drosophila brain. *Database* 2010, baq005 10.1093/database/baq005 20624714 PMC2911840

[DEV111054C37] Krogan, N. J., Cagney, G., Yu, H., Zhong, G., Guo, X., Ignatchenko, A., Li, J., Pu, S., Datta, N., Tikuisis, A. P. et al. (2006). Global landscape of protein complexes in the yeast Saccharomyces cerevisiae. *Nature* 440, 637-643 10.1038/nature04670 16554755

[DEV111054C38] Lécuyer, E., Yoshida, H., Parthasarathy, N., Alm, C., Babak, T., Cerovina, T., Hughes, T. R., Tomancak, P. and Krause, H. M. (2007). Global analysis of mRNA localization reveals a prominent role in organizing cellular architecture and function. *Cell* 131, 174-187 10.1016/j.cell.2007.08.003 17923096

[DEV111054C39] Lewellyn, L., Cetera, M. and Horne-Badovinac, S. (2013). Misshapen decreases integrin levels to promote epithelial motility and planar polarity in Drosophila. *J. Cell Biol.* 200, 721-729 10.1083/jcb.201209129 23509067 PMC3601364

[DEV111054C40] Li, X., Lobo, N., Bauser, C. and Fraser, M. Jr (2001). The minimum internal and external sequence requirements for transposition of the eukaryotic transformation vector piggyBac. *Mol. Genet. Genomics* 266, 190-198 10.1007/s004380100525 11683259

[DEV111054C41] Liao, G.-C., Rehm, E. J. and Rubin, G. M. (2000). Insertion site preferences of the P transposable element in Drosophila melanogaster. *Proc. Natl. Acad. Sci. USA* 97, 3347-3351 10.1073/pnas.97.7.3347 10716700 PMC16242

[DEV111054C42] Lichty, J. J., Malecki, J. L., Agnew, H. D., Michelson-Horowitz, D. J. and Tan, S. (2005). Comparison of affinity tags for protein purification. *Protein Expr. Purif.* 41, 98-105 10.1016/j.pep.2005.01.019 15802226

[DEV111054C43] Liu, J.-L. (2010). Intracellular compartmentation of CTP synthase in Drosophila. *J. Genet. Genomics* 37, 281-296 10.1016/S1673-8527(09)60046-1 20513629

[DEV111054C44] Lye, C. M., Naylor, H. W. and Sanson, B. (2014). Subcellular localisations of the CPTI collection of YFP-tagged proteins in Drosophila embryos. *Development* 141, 4006-4017. 25294944 10.1242/dev.111310 PMC4197698

[DEV111054C45] Lyne, R., Smith, R., Rutherford, K., Wakeling, M., Varley, A., Guillier, F., Janssens, H., Ji, W., McLaren, P., North, P. et al. (2007). FlyMine: an integrated database for Drosophila and Anopheles genomics. *Genome Biol.* 8, R129 10.1186/gb-2007-8-7-r129 17615057 PMC2323218

[DEV111054C46] Manhire-Heath, R., Golenkina, S., Saint, R. and Murray, M. J. (2013). Netrin-dependent downregulation of Frazzled/DCC is required for the dissociation of the peripodial epithelium in Drosophila. *Nat. Commun.* 4, 2790 10.1038/ncomms3790 24225841

[DEV111054C47] Marinho, J., Martins, T., Neto, M., Casares, F. and Pereira, P. S. (2013). The nucleolar protein Viriato/Nol12 is required for the growth and differentiation progression activities of the Dpp pathway during Drosophila eye development. *Dev. Biol.* 377, 154-165 10.1016/j.ydbio.2013.02.003 23416177

[DEV111054C48] Misra, S., Crosby, M. A., Mungall, C. J., Matthews, B. B., Campbell, K. S., Hradecky, P., Huang, Y., Kaminker, J. S., Millburn, G. H., Prochnik, S. E. et al. (2002). Annotation of the Drosophila melanogaster euchromatic genome: a systematic review. *Genome Biol.* 3, RESEARCH0083 10.1186/gb-2002-3-12-research0083 12537572 PMC151185

[DEV111054C49] Monier, B., Pélissier-Monier, A., Brand, A. H. and Sanson, B. (2010). An actomyosin-based barrier inhibits cell mixing at compartmental boundaries in Drosophila embryos. *Nat. Cell Biol.* 12, 60-65 10.1038/ncb2005 19966783 PMC4016768

[DEV111054C51] Morais-de-Sa, E., Vega-Rioja, A., Trovisco, V. and St Johnston, D. (2013). Oskar is targeted for degradation by the sequential action of Par-1, GSK-3, and the SCF(-)Slimb ubiquitin ligase. *Dev. Cell* 26, 303-314 10.1016/j.devcel.2013.06.011 23948254 PMC3744808

[DEV111054C52] Morin, X., Daneman, R., Zavortink, M. and Chia, W. (2001). A protein trap strategy to detect GFP-tagged proteins expressed from their endogenous loci in Drosophila. *Proc. Natl. Acad. Sci. USA* 98, 15050-15055 10.1073/pnas.261408198 11742088 PMC64981

[DEV111054C53] Narayanaswamy, R., Levy, M., Tsechansky, M., Stovall, G. M., O'Connell, J. D., Mirrielees, J., Ellington, A. D. and Marcotte, E. M. (2009). Widespread reorganization of metabolic enzymes into reversible assemblies upon nutrient starvation. *Proc. Natl. Acad. Sci. USA* 106, 10147-10152 10.1073/pnas.0812771106 19502427 PMC2691686

[DEV111054C54] Neumüller, R. A., Wirtz-Peitz, F., Lee, S., Kwon, Y., Buckner, M., Hoskins, R. A., Venken, K. J. T., Bellen, H. J., Mohr, S. E. and Perrimon, N. (2012). Stringent analysis of gene function and protein-protein interactions using fluorescently tagged genes. *Genetics* 190, 931-940 10.1534/genetics.111.136465 22174071 PMC3296255

[DEV111054C55] Ni, J., Liu, L.-P., Binari, R., Hardy, R., Shim, H.-H., Cavallaro, A., Booker, M., Pfeiffer, B. D., Markstein, M., Wang, H. et al. (2009). A Drosophila resource of transgenic RNAi lines for neurogenetics. *Genetics* 182, 1089-1100 10.1534/genetics.109.103630 19487563 PMC2728850

[DEV111054C56] Ni, J.-Q., Zhou, R., Czech, B., Liu, L.-P., Holderbaum, L., Yang-Zhou, D., Shim, H.-S., Tao, R., Handler, D., Karpowicz, P. et al. (2011). A genome-scale shRNA resource for transgenic RNAi in Drosophila. *Nat. Methods* 8, 405-407 10.1038/nmeth.1592 21460824 PMC3489273

[DEV111054C57] Noree, C., Sato, B. K., Broyer, R. M. and Wilhelm, J. E. (2010). Identification of novel filament-forming proteins in Saccharomyces cerevisiae and Drosophila melanogaster. *J. Cell Biol.* 190, 541-551 10.1083/jcb.201003001 20713603 PMC2928026

[DEV111054C58] Olesnicky, E. C., Bhogal, B. and Gavis, E. R. (2012). Combinatorial use of translational co-factors for cell type-specific regulation during neuronal morphogenesis in Drosophila. *Dev. Biol.* 365, 208-218 10.1016/j.ydbio.2012.02.028 22391052 PMC3642870

[DEV111054C86] Orchard, S., Ammari, M., Aranda, B., Breuza, L., Briganti, L., Broackes-Carter, F., Campbell, N. H., Chavali, G., Chen, C., del-Toro, N. et al. (2014). The MIntAct project--IntAct as a common curation platform for 11 molecular interaction databases. *Nucleic Acids Res.* 42, D358-D363 10.1093/nar/gkt1115 24234451 PMC3965093

[DEV111054C59] O'Sullivan, N. C., Jahn, T. R., Reid, E. and O'Kane, C. J. (2012). Reticulon-like-1, the Drosophila orthologue of the hereditary spastic paraplegia gene reticulon 2, is required for organization of endoplasmic reticulum and of distal motor axons. *Hum. Mol. Genet.* 21, 3356-3365 10.1093/hmg/dds167 22543973 PMC3392112

[DEV111054C60] Pechmann, S., Willmund, F. and Frydman, J. (2013). The ribosome as a hub for protein quality control. *Mol. Cell* 49, 411-421 10.1016/j.molcel.2013.01.020 23395271 PMC3593112

[DEV111054C61] Peng, H., Chung, P., Long, F., Qu, L., Jenett, A., Seeds, A. M., Myers, E. W. and Simpson, J. H. (2011). BrainAligner: 3D registration atlases of Drosophila brains. *Nat. Methods* 8, 493-498 10.1038/nmeth.1602 21532582 PMC3104101

[DEV111054C62] Pu, S., Wong, J., Turner, B., Cho, E. and Wodak, S. J. (2009). Up-to-date catalogues of yeast protein complexes. *Nucleic Acids Res.* 37, 825-831 10.1093/nar/gkn1005 19095691 PMC2647312

[DEV111054C63] Quinones-Coello, A. T., Petrella, L. N., Ayers, K., Melillo, A., Mazzalupo, S., Hudson, A. M., Wang, S., Castiblanco, C., Buszczak, M., Hoskins, R. A. et al. (2007). Exploring strategies for protein trapping in Drosophila. *Genetics* 175, 1089-1104 10.1534/genetics.106.065995 17179094 PMC1840052

[DEV111054C64] Redhouse, J. L., Mozziconacci, J. and White, R. A. H. (2011). Co-transcriptional architecture in a Y loop in Drosophila melanogaster. *Chromosoma* 120, 399-407 10.1007/s00412-011-0321-1 21556802

[DEV111054C65] Rees, J. S., Lowe, N., Armean, I. M., Roote, J., Johnson, G., Drummond, E., Spriggs, H., Ryder, E., Russell, S., St Johnston, D. et al. (2011). In vivo analysis of proteomes and interactomes using Parallel Affinity Capture (iPAC) coupled to mass spectrometry. *Mol. Cell. Proteomics* 10, M110.002386 10.1074/mcp.M110.002386 21447707 PMC3108830

[DEV111054C66] Rothbauer, U., Zolghadr, K., Muyldermans, S., Schepers, A., Cardoso, M. C. and Leonhardt, H. (2008). A versatile nanotrap for biochemical and functional studies with fluorescent fusion proteins. *Mol. Cell. Proteomics* 7, 282-289 10.1074/mcp.M700342-MCP200 17951627

[DEV111054C67] Ryder, E., Spriggs, H., Drummond, E., St Johnston, D. and Russell, S. (2009). The Flannotator - a gene and protein expression annotation tool for Drosophila melanogaster. *Bioinformatics* 25p, 548-549 10.1093/bioinformatics/btp012 19126575

[DEV111054C68] Schneider, M., Troost, T., Grawe, F., Martinez-Arias, A. and Klein, T. (2013). Activation of Notch in lgd mutant cells requires the fusion of late endosomes with the lysosome. *J. Cell Sci.* 126, 645-656 10.1242/jcs.116590 23178945

[DEV111054C69] Schwabe, T., Bainton, R. J., Fetter, R. D., Heberlein, U. and Gaul, U. (2005). GPCR signaling is required for blood-brain barrier formation in drosophila. *Cell* 123, 133-144 10.1016/j.cell.2005.08.037 16213218

[DEV111054C70] Schwartz, A. S., Yu, J., Gardenour, K. R., Finley, R. L. Jr and Ideker, T. (2009). Cost-effective strategies for completing the interactome. *Nat. Methods* 6, 55-61 10.1038/nmeth.1283 19079254 PMC2613168

[DEV111054C71] Sepp, K. J. and Auld, V. J. (1999). Conversion of lacZ enhancer trap lines to GAL4 lines using targeted transposition in Drosophila melanogaster. *Genetics* 151, 1093-1101. 10049925 10.1093/genetics/151.3.1093 PMC1460539

[DEV111054C72] Spradling, A. C., Stern, D., Beaton, A., Rhem, E. J., Laverty, T., Mozden, N., Misra, S. and Rubin, G. M. (1999). The Berkeley Drosophila Genome Project gene disruption project: single P-element insertions mutating 25% of vital Drosophila genes. *Genetics* 153, 135-177. 10471706 10.1093/genetics/153.1.135 PMC1460730

[DEV111054C73] Stanyon, C. A., Liu, G., Mangiola, B. A., Patel, N., Giot, L., Kuang, B., Zhang, H., Zhong, J. and Finley, R. L. Jr (2004). A Drosophila protein-interaction map centered on cell-cycle regulators. *Genome Biol.* 5, R96 10.1186/gb-2004-5-12-r96 15575970 PMC545799

[DEV111054C74] Strumpf, D. and Volk, T. (1998). Kakapo, a novel cytoskeletal-associated protein is essential for the restricted localization of the neuregulin-like factor, vein, at the muscle-tendon junction site. *J. Cell Biol.* 143, 1259-1270 10.1083/jcb.143.5.1259 9832554 PMC2133081

[DEV111054C75] Syed, M. H., Krudewig, A., Engelen, D., Stork, T. and Klambt, C. (2011). The CD59 family member Leaky/Coiled is required for the establishment of the blood-brain barrier in Drosophila. *J. Neurosci.* 31, 7876-7885 10.1523/JNEUROSCI.0766-11.2011 21613501 PMC6633149

[DEV111054C76] Thibault, S. T., Singer, M. A., Miyazaki, W. Y., Milash, B., Dompe, N. A., Singh, C. M., Buchholz, R., Demsky, M., Fawcett, R., Francis-Lang, H. L. et al. (2004). A complementary transposon tool kit for Drosophila melanogaster using P and piggyBac. *Nat. Genet.* 36, 283-287 10.1038/ng1314 14981521

[DEV111054C77] Timofeev, K., Joly, W., Hadjieconomou, D. and Salecker, I. (2012). Localized netrins act as positional cues to control layer-specific targeting of photoreceptor axons in Drosophila. *Neuron* 75, 80-93 10.1016/j.neuron.2012.04.037 22794263 PMC3398394

[DEV111054C78] Venken, K. J. T. and Bellen, H. J. (2005). Emerging technologies for gene manipulation in Drosophila melanogaster. *Nat. Rev. Genet.* 6, 167-178 10.1038/nrg1553 15738961

[DEV111054C79] Venken, K. J. T., He, Y., Hoskins, R. A. and Bellen, H. J. (2006). P[acman]: a BAC transgenic platform for targeted insertion of large DNA fragments in *D. melanogaster*. *Science* 314, 1747-1751 10.1126/science.1134426 17138868

[DEV111054C80] Venken, K. J. T., Schulze, K. L., Haelterman, N. A., Pan, H., He, Y., Evans-Holm, M., Carlson, J. W., Levis, R. W., Spradling, A. C., Hoskins, R. A. et al. (2011). MiMIC: a highly versatile transposon insertion resource for engineering Drosophila melanogaster genes. *Nat. Methods* 8, 737-743 10.1038/nmeth.1662 21985007 PMC3191940

[DEV111054C81] Voelker, R. A., Greenleaf, A. L., Gyurkovics, H., Wisely, G. B., Huang, S. M. and Searles, L. L. (1984). Frequent imprecise excision among reversions of a P element-caused lethal mutation in Drosophila. *Genetics* 107, 279-294. 17246216 10.1093/genetics/107.2.279 PMC1202322

[DEV111054C82] von Mering, C., Krause, R., Snel, B., Cornell, M., Oliver, S. G., Fields, S. and Bork, P. (2002). Comparative assessment of large-scale data sets of protein-protein interactions. *Nature* 417, 399-403 10.1038/nature750 12000970

[DEV111054C83] Will, C. L. and Luhrmann, R. (2001). Spliceosomal UsnRNP biogenesis, structure and function. *Curr. Opin. Cell Biol.* 13, 290-301 10.1016/S0955-0674(00)00211-8 11343899

[DEV111054C84] Yamamoto, S., Bayat, V., Bellen, H. J. and Tan, C. (2013). Protein phosphatase 1ss limits ring canal constriction during Drosophila germline cyst formation. *PLoS ONE* 8, e70502 10.1371/journal.pone.0070502 23936219 PMC3723691

[DEV111054C85] Zhao, T., Graham, O. S., Raposo, A. and St Johnston, D. (2012). Growing microtubules push the oocyte nucleus to polarize the Drosophila dorsal-ventral axis. *Science* 336, 999-1003 10.1126/science.1219147 22499806 PMC3459055

