# Supplementary material for: Analysis of the expression patterns, subcellular localisations and interaction partners of Drosophila proteins using a pigP protein trap library
Source: Development. 2014 Oct;141(20):3994–4005. doi: 10.1242/dev.111054 (PMC4197710; doi:10.1242/dev.111054)
Supplement: Supplementary Material [file supp_141_20_3994__index.html]

Supplementary Material 

# Analysis of the expression patterns, subcellular localisations and interaction partners of *Drosophila* proteins using a *pigP* protein trap library

## DEV111054 Supplementary Material

**Files in this Data Supplement:**

- **Supplementary Material**
