# Supplementary material for: Analysis of the expression patterns, subcellular localisations and interaction partners of Drosophila proteins using a pigP protein trap library
Source: Development. 2014 Oct;141(20):3994–4005. doi: 10.1242/dev.111054 (PMC4197710; doi:10.1242/dev.111054)
Supplement: Supplementary Material [file supp_141.20.3994_DEV111054.pdf]

Supplementary Materials and Methods

Constructs: The progenitors of the constructs used in the Cambridge Protein Trap Screen were as follows:

pGA, pGB and pGC are described in Morin et al (2001). These provided the *mini-white* gene and the artificial exon consisting of a 6His-EGFP gene flanked by splice donor and acceptor sequences from the *Mhc* gene. The artificial exon was flanked by *Nco*I and *Sal*I sites with variable length linkers outside these sites used to create the different splice frames.

p3E2.1 is the original *piggyBac* transposon plasmid from Fraser et al. (1995) and was used to provide the *piggyBac* sequences. Sequences required for trapping constructs were cloned into a unique *Hpa*I site within the *piggyBac* open reading frame.

pP{SVS-A} N473. A *P* element construct based on the original pGA vector (Morin et al, 2001). StrepTagII affinity tags were added at each end of YFP-venus by PCR and cloned back into the *Nco*I - *Sal*I sites of pGA.

In the descriptions below the following nomenclature is used:

PBac - These constructs only contain *piggyBac* transposon sequences.

PIGP - These constructs contain *P* element sequences from the original Morin constructs inserted into a unique *Hpa*I site in the *piggyBac* open reading frame.

{ } - Letters within the curled brackets, e.g. {SVS-} denote the type of affinity tag incorporated: F = 3xFLAG, S = StrepTagII, V = Venus YFP

Splice frames are denoted by 0, 1 or 2 and these designations are included in the names of the constructs. Morin et al (2001) designated these C, A and B respectively.

544 - PBac544{SVS-1} - Protein trapping construct based on the p3E1.2 *piggyBac* vector. The *mini-white* gene and Strep-tag/VenusYFP from pP{SVS-A}473 were inserted into the unique *Hpa*I site in the piggyBac vector. Only one splice frame was made with this vector, and it was used in small scale screen to produce the pilot NPSV protein trap lines CPTI-100034 to CPTI-100066.

566 - PIGP566{SVS-1} - Hybrid *piggyBac*/*P* element vector based on the p3E1.2 *piggyBac* plasmid. Used in a small scale screen to produce the protein trap lines CPTI-100000 to CPTI-100032. Splice frame 1

602 - PIGP602{SVS-1} - Hybrid piggyBac/P element trapping construct Identical to PIGP566{SVS-1} but with several potential cryptic splice sites in the *piggyBac* sequence mutated. This mutated backbone was used in all subsequent pigP constructs. It is not known whether these potential cryptic sites had any effect on trapping efficiency. Used in the high throughput screens with embryo sorter. Splice frame 1

681 - PIGP681{FSVS-1} - Hybrid *piggyBac/P*-element protein trapping vector containing 3xFLAG-StrepTag-Venus-StrepTag. Splice frame 1

754 - PIGP754{FSVS-0} - Hybrid *piggyBac/P* element protein trapping vector containing 3xFLAG-StrepTag-venus-StrepTag in splice frame 0

768 - PBac768{FSVS-0} - *piggyBac* protein trapping vector with 3xFLAG-StrepTag-Venus-StrepTag. This vector is similar to PIGP754{FSVS-0} but without the *P* element sequences. Splice frame 0.

769 - PBac769{FSVS-1} - *piggyBac* protein trap vector with 3xFLAG-Strep-Venus-Strep. Splice frame 1. Identical to PBac768{FSVS-0} but splice frame shifted by one base. Splice frame 1.

802 - PIGP802{SVS-2} - Hybrid *piggyBac/P* element protein trapping construct with StrepTag-Venus-StrepTag. This construct was made with a mutated ATG in the *P* element transposase to prevent the construct acting as an enhnacer trap (Buszczak et al., 2007; Quinones-Coello et al., 2007). Splice frame 2

803 - PIGP803{SVS-0} - Hybrid *piggyBac/P* element protein trapping construct with StrepTag-Venus-StrepTag in splice frame 0. Identical to PIGP802{SVS-2} but in splice frame 0.

806 - PBac806{LOX-SVS-2} - *piggyBac* protein trap construct without P element sequences. This was created because *P* element traps in this splice frame are prone to act as enhancer traps, giving in-frame fusions to the *P* element ORF (Buszczak et al., 2007; Quinones-Coello et al., 2007). Lox sites were incorporated to allow exchange of internal sequences by Recombinase Mediated Cassette Exchange (Oberstein et al., 2005). Splice frame 2

810 - PIGP810{FSVS-2} - *Hybrid piggyBac/P* element protein trapping construct with StrepTag-Venus-StrepTag. The initiating ATG of the *P* element transposase was mutated in this construct to prevent it acting as an enhancer trap. Splice frame 2.

Crossing schemes

Virginator stocks for J10 and J6 piggyBac transposase sources and donors.

1) ♂ w/Y, hs-hid; If/CyO; Sb/TM3, Ser **X** ♀ w; J6/SM1 ->
♂ w/Y, hs-hid; J6/CyO; (Sb) (Ser) **X** ♀ w; J6/CyO; (Sb) (Ser) ->

stock w/Y, hs-hid; J6/CyO (select against Sb and Ser)

2) ♂ w/Y, hs-hid; If/CyO; Sb/TM3, Ser **X** ♀ w; J10/TM2 ->
♂ w/Y, hs-hid; (If) (Cy); J10/TM3, Ser **X** ♀ w; (If) (Cy); J10/TM3, Ser ->

stock w/Y, hs-hid; J10/TM3 (select against If and Cy)

3) ♂ w/Y, hs-hid; If/CyO; Sb/TM3, Ser **X** ♀ w^{1118} ->
♂ w^{1118}/ Y, hs-hid; (If) (CyO) (Sb) (TM3, Ser) **X** ♀ w^{1118} ->

stock w^{1118}/ Y, hs-hid (select against If, Cy, Sb, Ser)

4) ♂ w/Y, hs-hid; If/CyO; Sb/TM3, Ser **X** ♀ w; MKRS, pBac{YFP} Sb ->

stock w/Y, hs-hid; CyO/+; MKRS, pBac{YFP}/TM3, Ser (select against Cy)

5) ♂ w/Y, hs-hid; If/CyO; Sb/TM3, Ser **X** ♀ w; CyO, pBac{YFP}/Sco ->

stock w/Y, hs-hid; CyO, pBac{YFP}/If; Sb/+ (select against Sb)

6) w; hs-hid, Sp/CyO **X** w; Gla/CyO, pBac, w^+ ->

stock w; hs-hid, Sp/CyO, pBac, w^+

7) w; hs-hid, Dr[1]/TM6B, Tb **X** w; TM6B/TM3, pBac, w^+ ->

stock w; hs-hid, Dr/TM3, pBac, w^+

8) double hs-hid J10 stock

♂ w/Y, hs-hid; J10/TM3, Ser **X** ♀ y w; Pr Dr/TM3, hs-hid, Sb ->

stock ♂ y w/Y, hs-hid; J10/TM3, hs-hid, Sb X ♀ y w/w; J10/TM3, hs-hid, Sb

9) ♀ killer to produce 'free' *w* ♂

♀ C(1)DX/y^+Y **X** ♂ w^{1118}/Y ->

♂ w^{1118}/y^+Y (♀ C(1)DX/Y die)

Screening from donors with dominant markers.

1) ch 4 insertion, with 50/50 mosaic eye pattern: 602.10a-11

G0 ♂ w/Y, hs-hid; J6 **X** ♀ w; pBac, w^+ 602.10a-11 -> heatshock
G1 ♀ w; J6/+;+/pBac **X** ♂ w -> Screen

2) 681 insertions on CyO

G0 ♂ w/Y, hs-hid; J10/TM3, hs-hid Sb **X** ♀ w; hs-hid, Sp/CyO, pBac, w^+ -> heatshock
G1 ♀ w; +/CyO, pBac, w^+; J10/+ **X** ♂ w -> Screen

3) 681 insertions on CyO and Gla

G0 ♂ w/Y, hs-hid; J10/TM3, hs-hid Sb **X** ♀ w; Gla, pBac, w^+/CyO, pBac, w^+ -> heatshock
G1 ♀ w; +/CyO or Gla, pBac, w^+; J10/+ **X** ♂ w -> Screen

4) 681 insertions on TM3, Sb Ser

G0 ♂ w/Y, hs-hid; J6/J6 **X** ♀ w; hs-hid, Dr/TM3, pBac, w^+ -> heatshock
G1 ♀ w; J6/+; +/TM3, pBac, w^+ **X** ♂ w -> Screen

5) 681 insertions on TM6B and TM3

G0 ♂ w/Y, hs-hid; J6/J6 **X** ♀ w; TM3, pBac, w^+/TM6B, pBac, w^+ -> heatshock
G1 ♀ w; J6/+; +/TM6C or TM3, pBac, w^+ **X** ♂ w -> Screen

6) 681 insertions on TM6B and TM3, using a-Tub pBac/T, CyO

G0 ♂ w/Y, hs-hid; a-Tub pBac-T, CyO/Sp **X** ♀ w; TM3, pBac, w^+/TM6B, pBac, w^+ -> heatshock
G1 ♀ w; a-Tub pBac-T, CyO /+; +/TM6C or TM3, pBac, w^+ **X** ♂ w -> Screen

'Free' w- ♂ produced by crossing C(1)DX/y+Y to w ♂

Affinity Purifications

Approximately 200 µg embryos representing all stages of embryonic development were homogenised and lysed in 1 ml base buffer (50 mM Tris, pH 7.5, 125 mM NaCl, 1.5 mM MgCl, 1 mM EDTA, 5% Glycerol, 0.4% Nonidet P-40 and 0.1% Tween 20 (modified from Veraksa *et al.,* 2005) on ice. The cleared supernatants (typically 20 mg) were then added to 100 µl prewashed resins; EZview ANTI- FLAG M2 Affinity Gel (Sigma) and *Strep*-Tactin sepharose (IBA). Native protein complexes were allowed to bind for 1 hour, followed by centrifugation at 2000 x g and

removal of the supernatant containing the unbound proteins. The resins were washed 3 times for 15 mins in 1 ml lysis buffer and the bound proteins eluted in 100 µl of elution buffer. Elution buffers were base buffer pH 7.4 containing either 100 µg/ml 3X FLAG peptide for FLAG resins, or 100 mM biotin for *Strep*-Tactin resins. Western blots were performed using anti-GFP to confirm the presence of tagged bait.

Sample preparation for MS/MS

Eluates were pooled and concentrated using a speedvac and then resuspended in SDS sample buffer and resolved for ~2 cm on 10% reducing SDS-PAGE gels. Analysis gels were Coomassie stained and four equal sized portions of the stained area were excised, washed, reduced in 2 mM DTT for 1 h at RT, alkylated in 10 mM Iodoacetamide for 30 min at RT and digested in the gel with 2 µg sequencing-grade porcine trypsin (Promega) overnight at 37ºC. Digests were concentrated using a speedvac and resuspended in 20 µl 0.1% formic acid.

Mass Spectrometry

All LC-MS/MS experiments were performed using a nano LC system (Eksigent) coupled to an LTQ Orbitrap mass spectrometer (Thermo Scientific, Waltham, MA). Separation of 5 µl peptides was performed initially on a precolumn (Presearch) at a flow rate of 10 µl/min. Concentrated peptides were then loaded onto a PepMap C18 reverse phase, 75 mm i.d., 15 cm analytical column (LC Packings) and eluted with an increasing acetonitrile gradient (5-35%) in 0.1 % formic acid at a flow rate of 300 nl/minThe columns were washed twice for 30 min between biological samples with 85% and 65% acetonitrile followed by 0.1% formic acid to minimise carryover. The Orbitrap was operated in data dependent mode, MS then 2x tandem MS (MS/MS) with a resolution of 30000, a dynamic exclusion of 0.3 Da m/z. Exclusion mass lists were employed to minimize known

abundant contaminants (Rees et al. 2011, Supplementary Fig. 7 online). Ions with charge states of 2+ and above were selected for fragmentation.

Protein identification

Resulting fragment masses (MS/MS) were searched using the MASCOT version 2.2 (Matrix Science) search engine against an in house database comprising FlyBase *D. melanogaster* genome (version 5.9) plus the FASTA sequence for YFP to confirm the presence of the tagged protein. Parameters included 2 missed cleavages and methionine oxidation variable- and carboxymethyl-cysteine fixed-modifications. The decoy database option was selected to calculate the protein false discovery rate (FDR) and the cut-off was set to 5%. Proteins from negative controls and test samples were compared using ProteinCenter™ (Thermo Fisher).

Interaction Evaluation

The protein-protein interactions were evaluated using a Generalized Iterative Scaling-Maximum Entropy supervised machine learning approach. Positive and a negative training sets were constructed based on the CYC08 dataset mapped by homology to *D. melanogaster* proteins (Pu et al., 2009). The positive training set consisted of 823 binary protein associations, after removing histone and ribosomal protein complexes. The negative set was obtained by random sampling the set of proteins for protein pairs not published to have any direct physical (e.g. two hybrid, coimmunoprecipitation) or genetic interaction retrieved from FlyMine v34 (Lyne et al., 2007). The final result is a score between 0 and 1 where 0 corresponds to a very unlikely protein association and 1 to a very likely association. The score of 0.5 corresponds to associations for which the annotations were too sparse or non-specific to draw conclusions. The interactions were independently evaluated by examining whether they had been also observed in other datasets, such as yeast two hybrid screens, affinity purifications and genetic interaction screens using the FlyMine

database (v15). Since yeast two hybrid screens detect direct protein-protein interactions, whereas affinity purifications identify proteins that are present in the same complex, we also counted indirect yeast two hybrid interactions, in which the bait and prey proteins are linked through one intermediate protein.

Supplementary References:

Buszczak, M., Paterno, S., Lighthouse, D., Bachman, J., Planck, J., Owen, S., Skora, A. D., Nystul, T. G., Ohlstein, B., Allen, A. et al. (2007). The Carnegie protein trap library: a versatile tool for *Drosophila* developmental studies. *Genetics* **175**, 1505-1531.

Fraser, M. J., Cary, L., Boonvisudhi, K. and Wang, H. G. (1995). Assay for movement of Lepidopteran transposon IFP2 in insect cells using a baculovirus genome as a target DNA. *Virology* **211**, 397-407.

Lyne, R., Smith, R., Rutherford, K., Wakeling, M., Varley, A., Guillier, F., Janssens, H., Ji, W., McLaren, P., North, P. et al. (2007). FlyMine: an integrated database for *Drosophila* and *Anopheles* genomics. *Genome Biol.* **8**, R129.

Morin, X., Daneman, R., Zavortink, M. and Chia, W. (2001). A protein trap strategy to detect GFP-tagged proteins expressed from their endogenous loci in *Drosophila*. *Proc. Natl. Acad. Sci. USA* **98**, 15050-15055.

Oberstein, A., Pare, A., Kaplan, L. and Small, S. (2005). Site-specific transgenesis by Cre-mediated recombination in Drosophila. *Nat. Methods* **2**, 583-585.

Pu, S., Wong, J., Turner, B., Cho, E. and Wodak, S. J. (2009). Up-to-date catalogues of yeast protein complexes. *Nucleic Acids Res.* **37**, 825-831.

Quinones-Coello, A. T., Petrella, L. N., Ayers, K., Melillo, A., Mazzalupo, S., Hudson, A. M., Wang, S., Castiblanco, C., Buszczak, M., Hoskins, R. A. et al. (2007). Exploring strategies for protein trapping in *Drosophila*. *Genetics* **175**, 1089-1104.

Rees, J. S., Lowe, N., Armean, I. M., Roote, J., Johnson, G., Drummond, E., Spriggs, H., Ryder, E., Russell, S., St Johnston, D. et al. (2011). In vivo analysis of proteomes and interactomes using Parallel Affinity Capture (iPAC) coupled to mass spectrometry. *Mol. Cell Proteomics* **10**, M110 002386.

Veraksa, A., Bauer, A. and Artavanis-Tsakonas, S. (2005). Analyzing protein complexes in Drosophila with tandem affinity purification-mass spectrometry. *Dev. Dyn.* **232**, 827-834.

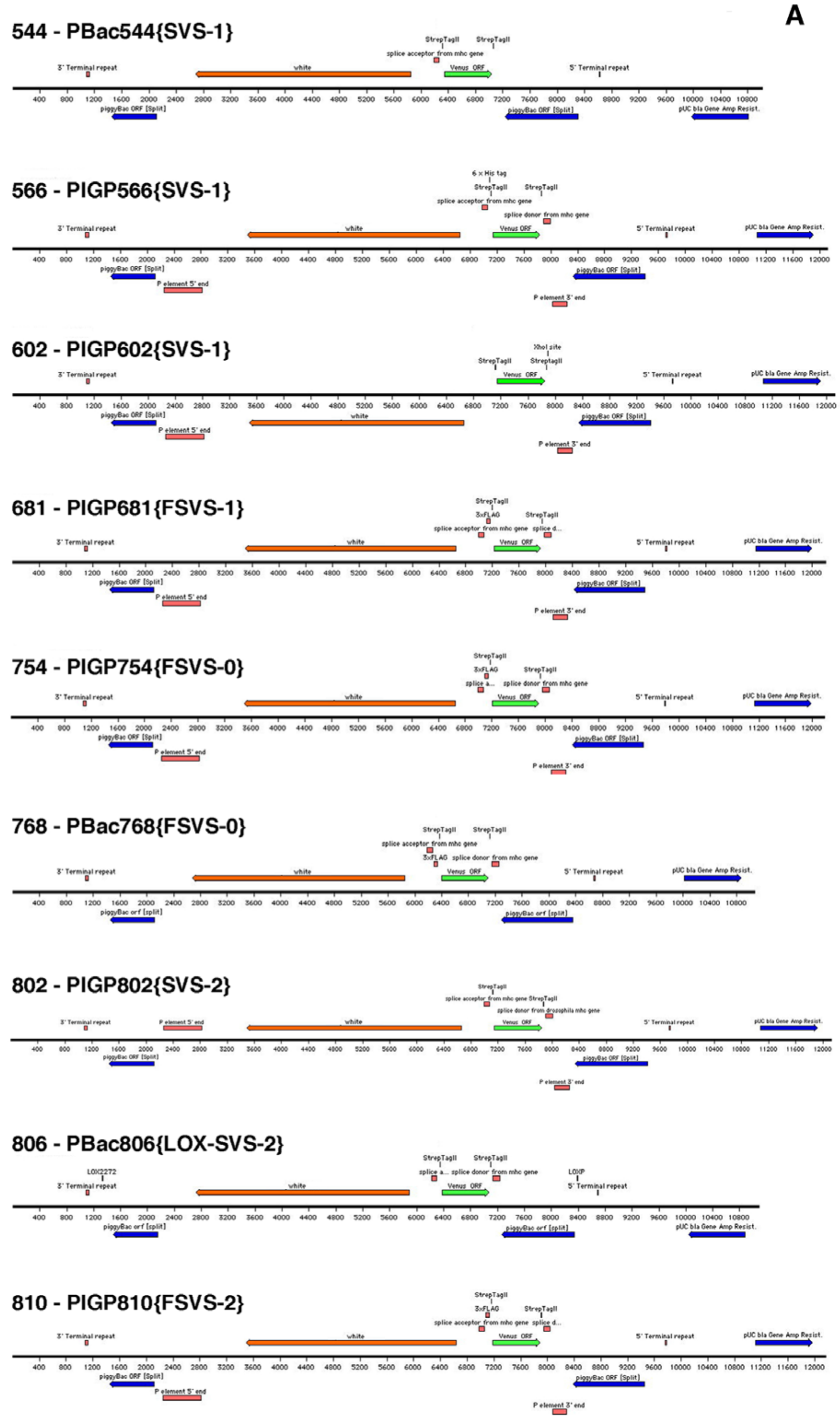

* 769 - PBac769{FSVS-1} is identical to 768 but in splice frame 1.
803 - PIGP803{SVS-0} is identical to 802 but in splice frame 0.

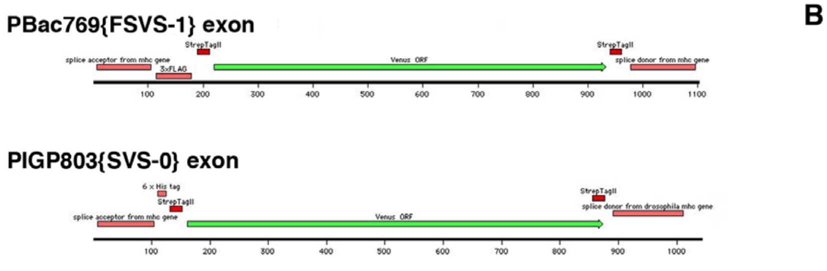

Supplementary Fig. S1: (A) Graphical representation of the protein trap vectors used. (B) Graphical representation of the two exon sequences used in the protein trap vectors.

Table S1: Summary of the protein trap screen. Donor name identifies the individual screen using the shorthand designation for the vector employed and the donor chromosome. Passed size gate = number of embryos selected by the COPAS time of flight selection. Passed sort gate = number of embryos selected as GFP positive by the COPAS select. %Sorted = the fraction of GFP positive embryos. Rescreened = number of surviving adults from GFP embryos subsequently rescreened. YFP+ = number of lines with YFP positive embryos after rescreening on the COPAS.

Download Table S1

Table S2: Excel file of CPTI lines. CRTI line = insertion stock designation; Gene name = Flybase Gene name; Gene CG = Flybase CG identifier; Subcellular localisation = annotated subcellular locations where available; Tissue Expression comments = notes on appearance of GFP expression; Chr -= chromosome; Location = release 5.65 genomic coordinates; Interaction data = mass-spectrometry data available; Viability = overall phenotypes (l = lethal, v = viable, sl = semi-lethal, fs = female sterile. Inframe = lines scored as in-frame insertions by initial iPCR screen. Kyoto = lines available from Kyoto stock centre. Notes = comments or warnings about individual lines. Evidence = for lines initially scored as out of frame, indication of whether proteomics or YFP localisation support protein trap. Y = yes, MS = peptides identifies by mass spectrometry, P = possible protein trap, not considered as positive in the manuscript, ? = insufficient data.

Download Table S2

Table S3: CPTI lines confirmed with 5' or 3' RACE

CPTI Line	RACE Result
CPTI-000016	*Megalin*
CPTI-000023	*sgg*
CPTI-000077	*HDAC4*
CPTI-000113	*Cad87A*
CPTI-000155	*glec*
**CPTI-000218**	***Meltrin***
**CPTI-000298**	***CG8920***
CPTI-000426	*Dek*
CPTI-000616	*ed*
**CPTI-001403**	***cher***
**CPTI-002064**	***PH4αEFB***
CPTI-002618	*babos*
CPTI-002831	*Pop2*
CPTI-003588	*piwi*
CPTI-003738	*Myo10A*
CPTI-100067	*obst-E*

Table S4: Genes with protein traps. Lists of protein traps identified in Cambridge and FlyTrap collections (All), found in both (Common), and unique to each collection.

Download Table S4

Table S5: Excel file of gene ontology enrichments. GO term enrichments for Biological process, Cellular component and Molecular function, corrected p-values and number of genes annotated with each term are provided.

Download Table S5
